# Lossless and Lossy Characterization of the State of Perturbed Anharmonic Diatomics: An Information-Theoretic Compaction of Quantum Dynamics

**DOI:** 10.1021/acs.jctc.6c00025

**Published:** 2026-02-18

**Authors:** James R. Hamilton, Raphael D. Levine

**Affiliations:** † The Fritz Haber Center for Molecular Dynamics, Institute of Chemistry, 26742 The Hebrew University of Jerusalem; Jerusalem 91904, Israel; ‡ Department of Molecular and Medical Pharmacology, David Geffen School of Medicine and Department of Chemistry and Biochemistry, University of California, Los Angeles, California 90095, United States

## Abstract

A lossless, exact compaction of the time-evolved state of the quantum dynamical system of a perturbed anharmonic molecule is demonstrated using dynamical symmetries. The density matrix of the anharmonic molecule is a linear combination of these symmetries, and it remains so as a time-dependent perturbation is applied. Accurate, unitary-but-approximate, and thereby irreversible compaction is further shown using fewer symmetries, and the fidelity of this lossy compaction is quantified. Perturbations are typically linear in the operators of a Lie algebra. For a Hamiltonian that is also linear, one knows well how to reversibly compact the state of a dynamical system. However, anharmonic vibrations have a finite number of unequally spaced energy levels, and a good description of their spectra typically requires an algebraic-type Hamiltonian that is bilinear in the operators of a Lie algebra. For a bilinear Hamiltonian we show how a matrix-based approach allows us to compact both the populations and the coherences, either exactly reversibly or inexactly irreversibly, with fewer symmetries. A forced Morse oscillator is used as an explicit analytical and numerical example covering the entire range of dynamics from the sudden to the adiabatic limits.

## Graphical abstract

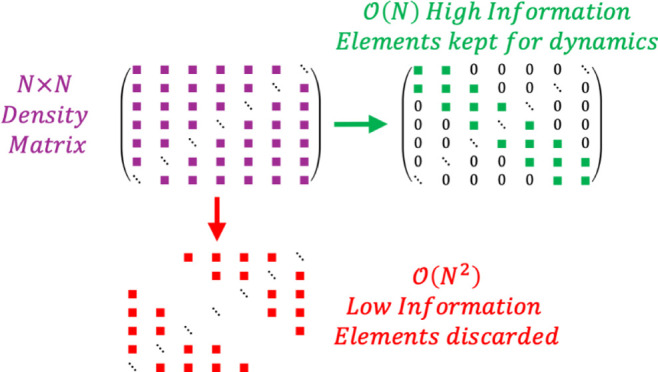

## Introduction

This paper is about the characterization of the state of a dynamical system, by which we mean specifying a (minimal) set of operators that suffice to characterize how the system will evolve in time. The expectation values of these operators are to be referred to here as the variables of the state. The system we discuss is that of an isolated anharmonic oscillator perturbed by a transient force. We aim to provide explicit characterizations of the state of the system, both exact and approximate, at any point in time, starting from an initially unperturbed oscillator and ending with the state after the perturbation is over.

As will be explained in the main sections of the text, the density matrix of the system can be constructed from sets of operators, which will become known as constraints. These operators can either be time dependent, or time independent with an associated time dependent coefficient. The actual variables which characterize the state are the expectation values of these operators over the density matrix of the system. A characterization of the state is a compaction when it requires (far) fewer variables than might appear to be needed. It is convenient to use Lie algebraic operators, but we only use the very elementary and introductory aspects of Lie algebras.^1,2^ A practical point is that because anharmonic oscillators have only a finite number of bound states, one can effectively represent the Lie algebraic operators as finite matrices. We need that the matrices satisfy the same commutation relations as the abstract Lie operators. What we want is an explicit prescription for building the variables of the state during and after the transient perturbation.

In^3^ we demonstrated the compaction of the dynamics of the system of an anharmonic Morse oscillator, perturbed by an extremely brief, sudden force. The sudden approximation of the Hamiltonian, the validity of which was demonstrated for a very fast perturbation, was linear and hence closed with an appropriate Lie algebra. In this work, we will demonstrate the compaction of the dynamics of the system of the forced Morse Oscillator, without any restriction on the duration of the perturbing force. We will therefore compact the dynamics of a system described by a bilinear Hamiltonian which is accurate for the whole durational range of possible perturbations, from sudden to adiabatic. In thermodynamics there can be many paths leading from one state to another. Most of these paths are irreversible. Here we primarily deal with a particular reversible path as specified by nonrelativistic quantum mechanics. This means that the state is well-defined at any point in time along the path. Does this sound like thermodynamics way out of equilibrium? Yes, it does. But this is not what we mean to highlight. What we want is an explicit prescription for building the variables of the state during and after the transient perturbation.

We will propose procedures for both exact, lossless, reversible compaction and lossy, irreversible compaction. We use the concepts of lossless and lossy compression in their standard information theoretic sense (see, for example, ref 4). Lossless compression is a method for compacting data which entails no loss of information. Mathematically, lossless compression reduces the size of a data object, for example by removing redundancy, without affecting its entropy. Lossless compression is reversible; the original data can be fully recovered from the compacted data. Lossy compression reduces the size of the data object with some loss of information, which has a consequent effect on its entropy. Lossy compression is irreversible as the information lost is permanently discarded. We use the concept of lossy compression in quantum information theory to mean the removal of constraints from the surprisal of the density matrix, without significantly altering its populations. As will be discussed, the concept of fidelity (the fidelity of the compacted surprisal density matrix to the original) is introduced as the measure of significance.

If there is structure in the problem, compaction is possible in principle, although not necessarily in practice. Typically, a written text can be compressed by taking advantage of the structure of the language, but a text of randomly generated characters cannot be compacted. The mathematical equivalent of the statement that the system has structure, is that the entropy is below its global maximum. In exact, unitary, reversible dynamics the entropy of the state is unchanged with time.

There is a highly developed quantum information theory that deals, in particular, with data compression, see for exampleref^5^. In such terms we wish to reliably transmit the results of the dynamics through a noiseless channel. Then, by Schumacher’s theorem,^6^ we need a capacity that is equal or exceeds the entropy of our system. However, in this paper we do not deal with the needed channel or storage capacity, but rather with the properties of the source itself. As will be discussed, quantum information theory also provides the measure of the quality of the compaction.

An exact characterization of the state at all times is formally easy to state: An exact characterization of the initial state plus quantum dynamics can determine the explicit exact characterization at all future times. This may seem like a tautology, but it is not. As we shall see, unitary quantum dynamics can lead to a lossy characterization. An explicit construction of the exact variables of the state is possible but the method needs a careful derivation. The purely formal statement is that the exact variables of the state are time dependent constants of the motion.^7–14^ Seemingly, constructing such variables is intractable for systems such as the anharmonic oscillator, which are defined by *bi*linear Hamiltonians. We shall show how the same methods that work well for constructing time dependent constants of the motion for linear Hamiltonian systems can be made to work, albeit at a price, for realistic bilinear Hamiltonian systems as well.

Our technical difficulties arise because we mean to explicitly allow the vibrations to be anharmonic. The preliminary task is what to specify as an initial, unperturbed, state of the oscillator. One option, commonly used by theorists, is to start the dynamics from a single, pure, quantum state, often the lowest energy state. Experiments often use mixtures of pure states. A more general initial state can be a coherent superposition of pure states. One can also think of an entangled state where the vibrations are entangled with electronic states, as can be the case for an attosecond perturbation.^15,16^ The algebraic approach that we will discuss is very much suited to specify entangled states. In this paper we restrict our attention to superpositions of states, coherent or incoherent. We shall use the information theory based maximal entropy formalism^17–19^ in its quantum mechanical version^20–26^ to specify an initial state. In the limit, this allows also for a pure state. We know of no other overall prescription for specifying a generic unperturbed state. Our first requirement is a Hamiltonian that allows a practical implementation of a quantum maximum entropy formalism. Not a problem if all we need is the initial state, but one actually needs a time dependent Hamiltonian, with a perturbing part, which can span a dynamical range from an adiabatic to a sudden^3,27–29^ perturbation. A variety of model anharmonic potentials can be cast in an algebraic form where the Hamiltonian of the unperturbed molecule is bilinear in the generators of a Lie algebra.^30–32^ Model perturbations are often linear in the generators. If also the Hamiltonian is linear in the generators, we have a well-developed machinery for implementing the dynamics.^7,9,10,33–38^ This is because the commutator of the Hamiltonian with the perturbation is closed, meaning it is a linear combination of the generators. Unfortunately, this is not our case. When the Hamiltonian of the unperturbed system is bilinear, one typically has an infinite perturbation series. But by going over to a matrix point of view, we discuss a way out. This allows us to offer both an exact and an approximate solution with the key advantage that both offer a significant compaction of the dynamics of a perturbed anharmonic oscillator.

Other anharmonic systems such as the electronic states of a hydrogen-like atom also have a bilinear Hamiltonian. The approach we discuss is equally useful for characterizing and compacting the dynamic response to perturbations for such systems. It is a general approach for bilinear Hamiltonians.

Our analysis can be cast in purely dynamical terms, see refs 7–13, 39–41 . However, looking at the dynamics in terms of the information theoretic maximal entropy form of the density matrix^20–22,26,42^ provides a clear and persuasive interpretation. See in particular^43^ for applications in optical spectroscopy. Exact compaction is equivalent to providing a set of operators, the constraints, such that the state of maximal entropy subject to these constraints is the exact dynamical state of the system. What is new in this paper is that we show that one can specify such a finite set also for strictly anharmonic systems. Such systems have a finite set, say *K* in number, of bound states. The Hilbert space is therefore *K*-dimensional and a general Hermitian operator can be represented by a *K* × *K* Hermitian matrix, or by *K* ^2^ real numbers. We show that in general it takes *K* constraints to exactly compact our system. If we use fewer operators for the compaction, some information will be lost, and the state of maximal entropy will necessarily have a higher entropy—the compaction will be lossy. The information lost is that represented by the constraints which are not included in the compaction. The increase of the entropy quantifies this loss of information. This is equivalent to saying the larger entropy represents the greater uncertainty there is about the system after these constraints have been removed. By analogy to the Schumacher noiseless channel coding theorem, we show that a lossy compaction, when generated by a unitary time evolution operator, is guaranteed irreversible.

To remain concrete, we illustrate our compaction procedures with applications to the well-known Morse oscillator.^30,44–50^ We provide numerical results for the system of a perturbed anharmonic Morse oscillator, with a realistic value of *K* = 33 bound states. The results we show for lossy compaction actually improve in fidelity when the number of bound states increases. We also provide purely analytical explicit results for very low *K* (Supporting Information Section 6). Analytical expressions for higher values run to pages of mathematics and therefore have to be encoded algorithmically. The details of these algorithms are explained in the (Supporting Information (Section 2). Our programs are available upon request.

The technical discussion begins with a preamble about the Morse oscillator. This is followed by a section on lossless compaction in principle, and then a section on a lossless compaction in practice. Finally, there is a section on lossy compaction, including an explicit numerical example.

### A Technical Preamble

We introduce the essential issue through an explicit example. A typical Hamiltonian that is bilinear in the generators of a Lie algebra is the Hamiltonian for the bound states of a Morse oscillator. It can be written in terms of angular momentum operators^3,10,48,51^

1 $${Ĥ}_{0}=A\left({{Ĵ}_{x}}^{2}+{{Ĵ}_{y}}^{2}\right)=A\left({Ĵ}^{2}-{{Ĵ}_{z}}^{2}\right)$$

The hat denotes an operator. A is the anharmonicity parameter. *Ĵ* is the total angular momentum with components ${Ĵ}_{x},{Ĵ}_{y}$ and ${Ĵ}_{z}$ , that satisfy the familiar angular momentum commutation relations $\left[{Ĵ}_{z},{Ĵ}_{y}\right]=-i{Ĵ}_{x}$ , $\left[{Ĵ}_{z},{Ĵ}_{x}\right]=i{Ĵ}_{y}$ and $\left[{Ĵ}_{y},{Ĵ}_{x}\right]=-i{Ĵ}_{z}$ . The three components are clearly closed under commutation. One can also replace ${Ĵ}_{x}\text{and}{Ĵ}_{y}$ by the linear combinations the ${Ĵ}_{+}$ and ${Ĵ}_{-}$ operators that induce one quantum incremental increases or decreases in the states of the oscillator, as such they are creation and annihilation operators, respectively. ${Ĵ}_{x}=\left({Ĵ}_{+}+{Ĵ}_{-}\right)/2$ and ${Ĵ}_{y}=-i\left({Ĵ}_{+}-{Ĵ}_{-}\right)/2$ . The set $\left\{{Ĵ}_{+},{Ĵ}_{-},{Ĵ}_{z}\right\}$ is also closed under commutation, $\left[{Ĵ}_{+},{Ĵ}_{-}\right]=2{Ĵ}_{z}$ and $\left[{Ĵ}_{z},{Ĵ}_{+}\right]={Ĵ}_{+}$ and $\left[{Ĵ}_{z},{Ĵ}_{-}\right]=-{Ĵ}_{-}$ .

We take a model transient time dependent perturbation by analogy to the harmonic oscillator limit, namely a coupling of a vibrational state only to its near neighbors

2 $$\mathrm{V̂}\left(t\right)=f\left(t\right)\left({Ĵ}_{+}+{Ĵ}_{-}\right)=2f\left(t\right){Ĵ}_{x}$$

This coupling has been often used before in studies of energy transfer to a Morse oscillator.^3,45,51–57^

The problem one faces doing dynamical calculations for this system is that to solve algebraically the time-dependent Schrödinger equation for the wave function, or the von Neumann equation of motion for the density matrix, one needs to commute the Hamiltonian of the unperturbed system with the perturbation. This is not a problem if the Hamiltonian of the oscillator is linear in the generators. The set of generators will be closed as they are members of a Lie algebra. For the bilinear form of ${Ĥ}_{0}$ , the commutator with the perturbation is bilinear in the generators. The next commutator with the bilinear ${Ĥ}_{0}$ will be trilinear, and so forth. To breakthrough, we explicitly recognize that we want to solve for some specific but generic oscillator of a given, but arbitrary, well depth. Equivalently, we want to solve for a given number, say 2*j* + 1, of bound states.[Fn fn1] An example of where the number of variables of state can be dramatically reduced, is for the dynamical system of a Morse oscillator forced by a perturbation in the sudden limit. We previously showed analytically that, when the sudden approximation is used, two variables of state exactly suffice for compacting a Morse^3^ oscillator with a thermal initial state.

There are quite a number of analytical potentials for an anharmonic molecule. Many require more than two parameters to fit the properties of a particular molecule. The empirical success of the “law of corresponding states”^58^ suggests that already with two parameters one can achieve a realistic account of an anharmonic spectrum. A spectroscopist will say that these are just the vibrational frequency and the anharmonicity, *Aj* and *A* in the case of the Morse oscillator.

Besides the Morse Hamiltonian of eq 2 other potentials for a vibrational motion are specifiable by two parameters and can be represented by Hamiltonians bilinear in the generators of an algebra. These include Eckart, Pöschl-Teller, Rosen-Morse and the Kratzer potential.^59^ For such potentials one can, as we do in this work, use the matrix representation of the Hamiltonian in the basis of the bound states.

Again, taking the Morse oscillator as an example, it has bound states |*j*,*m*_*j*_⟩that for a given *j* are orthonormal eigenstates of **H**_0_. In this basis the operators are represented as matrices, so from now on they will be exhibited as boldface characters. For example, **J** ^2^|*j*,*m*_*j*_⟩ = *j*(*j* + 1)|*j*,*m*_*j*_⟩ so that *j* is the total angular momentum quantum number and *m*_*j*_, which spans the range from −*j* to *j*, is the total angular momentum projection quantum number. The unperturbed Hamiltonian in this basis is a diagonal matrix of dimensions (2*j* + 1) × (2*j* + 1). In a slight abuse of notation, these matrices can be written in terms of operators. For a specific value of *j* we have $H_{0}=\sum_{m_{j}=-j}^{j}E_{m_{j}}|j,m_{j}\rangle \langle j,m_{j}|$ . The component of **J** along the *z* axis is equally diagonal

3 $$J_{z}=\sum_{m_{j}=-j}^{j}m_{j}|j,m_{j}\rangle \langle j,m_{j}|$$

The raising and lowering operators are super- and sub-diagonal

4 $$J_{+}=\sum_{m_{j}=-j}^{j}{\left(j\left(j+1\right)-m_{j}\left(m_{j}+1\right)\right)}^{1/2}|j,m_{j}+1\rangle \langle j,m_{j}|$$

5 $$J_{-}=\sum_{m_{j}=-j}^{j}{\left(j\left(j+1\right)-m_{j}\left(m_{j}-1\right)\right)}^{1/2}|j,m_{j}-1\rangle \langle j,m_{j}|$$

Any other operator is similarly represented as a matrix of dimensions (2*j* + 1) × (2*j* + 1).

A basis of *K* bound states is available for all other algebraic Hamiltonians. An operator in the space of the bound states is a *K* × *K* matrix of the same dimensions as that of the Hamiltonian.

The basic concepts of quantum information theory will now be introduced, followed by a discussion of two measures by which the information lost in the compaction can be quantified, namely the entropy difference, Δ*S*, and the fidelity. The density matrix in maximum entropy form is

6 ρ(t)=exp(−I(t))

The quantum mechanical equivalent of the classical surprisal,^7,60^ the exponent of **ρ**, is written as the sum of constraints, {**X**_*k*_}, and Lagrange parameters, {λ_*k*_}­

7 $$I=\sum_{k}\lambda_{k}X_{k}$$

Note, the number of terms needed in the summation depends on the particular problem. One can express the surprisal as a sum of time dependent constraints with time independent Lagrange parameters, $I\left(t\right)=\sum_{k}\lambda_{k}X_{k}\left(t\right)$ , or, alternatively, as a sum of time independent constraints with time dependent Lagrange parameters $I\left(t\right)=\sum_{k}\lambda_{k}\left(t\right)X_{k}$ . The latter requires that we are able to express the time dependent constraints as linear combinations of time-independent operators. The quantifying measure of the uncertainty about a quantum system (defined by a density matrix **ρ**) in quantum information theory is the von Neumann entropy,^61^ *S* = −Tr­[**ρ**·ln­(**ρ**)].

For a density matrix of maximal entropy, ln**ρ**(*t*) = −∑_*k*_λ_*k*_ **X**_*k*_, the expectation value of the constant of motion, ⟨**X**_*k*_(*t*)⟩, is constant. Further, ⟨**X**_*k*_(*t*)⟩ has the same value when computed over the exact density matrix as over the density matrix of maximal entropy. If **ρ** ^A^ is an exact description of a system, calculated by numerical integration of the Liouville von-Neumann equation of motion for the density matrix (eq 30), or some other method, and **ρ** ^B^ is a density matrix of maximum entropy subject to the same constraints as **ρ** ^A^, the entropy difference between the two is

8 $$\Delta S=Tr\left[\rho^{A}\cdot \ln\left(\rho^{A}/\rho^{B}\right)\right]$$

One can validate the claim of lossless compression using entropy difference as a measure. Δ*S* must vanish if the compaction is exact because it is the difference in the entropies of **ρ** ^A^ and **ρ** ^B^.

Fidelity is an alternative measure of how much information is preserved after the medium in which it is encoded has undergone some process. Fidelity measures “how close” the information is after the process to what it was before.

In quantum information theory, the fidelity between two states **ρ** ^A^ and **ρ** ^B^ is defined as (ref 5 page 409) $F\left(\rho^{A},\rho^{B}\right)=tr\left(\sqrt{\sqrt{\rho^{A}}\rho^{B}\sqrt{\rho^{A}}}\right)$ . The fidelity ranges from 0 to 1. A fidelity of 1 means the information is perfectly preserved, it is easy to see that *F*(**ρ** ^A^,**ρ** ^A^) = 1. If **ρ** ^B^ is an exact, lossless compression of **ρ** ^A^,F­(**ρ** ^A^,**ρ** ^B^) = 1.

To repeat, for exact lossless compression, Δ*S* = 0 and *F* = 1. In an actual computation, these perfect equalities will not be met because of inevitable numerical errors in either way of propagating the density matrix in time.

Note that, as both the entropy difference and the fidelity result from operations on the time dependent density matrices, they too are implicitly time dependent, Δ*S*(*t*) and *F*(**ρ** ^A^(*t*),**ρ** ^B^(*t*)). Generally it is tidier not to state this time dependence explicitly.

As we will discuss, in a lossy compaction Δ*S* remains as the difference in the entropies of **ρ** ^A^ and of **ρ** ^B^, but it is finite and positive because the entropy of **ρ** ^B^ is not maximally lowered. Both validity measurements, Δ*S* and *F*, will concur about how much information is removed by lossy compression.

### Toward Lossless Compaction in Principle

Using the indices *i* or *k* to enumerate the bound states, we make the trivial but useful statement that there is a closed Lie algebra made up of the *K* ^2^ operators that are the outer products ${Ê}_{ik}\equiv \left|i\rangle \langle k\right|$ where the eigenstates indices *i* and *k* span the range 1 to *K*. Closure is easy to check: [|*i*⟩⟨*k*|,|*l*⟩⟨*m*|] = δ_*jl*_|*i*⟩⟨*m*|−δ_*mi*_|*l*⟩⟨*k*|. Switching to matrix notation, we have, therefore, a closed Lie algebra of *K* ^2^ matrices, {**E**_*ik*_}. The set of matrices, {**E**_*ik*_}, are the Gelfand matrices. The element of **E**_*ik*_ in row *i*, column *j* is equal to one, and all the other elements are equal to zero. Every matrix **M** of interest is a linear combination of these matrices, **M** = ∑_*i*,__*k*_^*K*^ *m*_*ik*_ **E**_*ik*_.

Of course, this equation simply says that *m*_*ik*_ use the *ik* matrix element of the matrix **M**. The variables of state are now the *K* ^2^ expectation values of the matrices {**E**_*ik*_}, the {⟨**E**_*ik*_⟩}, or any invertible linear transformation thereof. In general, these expectation values will be complex. In the case where **M** is a Hermitian matrix, the expectation values of the matrix will be constituted of *K* ^2^ real numbers. This is because any expectation value of a matrix in the Liouville space of observables is a linear combination of the *K* ^2^ expectation values of the generators of the Lie algebra spanned by the **E**_*ik*_^′^ *s*. We have an exact representation of the density matrix, but so far not a compaction. We have *K* ^2^ variables of state that fully describe the dynamics in the space of bound states.

The density matrix **ρ**(*t*) is Hermitian and satisfies Liouville’s theorem, it can therefore be diagonalized at any time *t*

9 $$\rho \left(t\right)=\sum_{k=1}^{K}p_{k}P_{k}\left(t\right)$$

in the absence of degeneracies the **P**_*k*_(*t*)^′^s are one-dimensional projectors. We aim to contrast the general expression eq 9 that is valid for any density matrix with the special form

10 $$\rho \left(t\right)=U\left(t\right)\rho \left(0\right)U^{†}\left(t\right)=\sum_{k=1}^{K}\rho_{k}{U\left(t\right)P}_{k}\left(0\right)U^{†}\left(t\right)$$

Where **U**(*t*) is a unitary time evolution matrix for the revolution generated by **H**_0_ + **V**(*t*). In eq ([Disp-formula eq10]) the density matrix **ρ**(*t*) evolved from a stationary initial state, as shown. The *K* time dependent matrices **P**_*k*_(*t*)­are projectors which losslessly compact the state **ρ**(*t*) of the system. Further, the **P**_*k*_(*t*), like **ρ**(*t*), are dynamical symmetries: Heisenberg picture operators that move backward in time.^11,62,63^ They are the time dependent constants of the motion that correspond to the *K* projection operators that compact the stationary initial state:

$$P_{k}\left(t\right)={U\left(t\right)P}_{k}\left(0\right)U^{†}\left(t\right)={U\left(t\right)E}_{kk}U^{†}\left(t\right)={U\left(t\right)\left|k\rangle \langle k\right|U}^{†}\left(t\right)\equiv \left|k,t\rangle \langle k,t\right|$$

Here |*k*,*t*⟩ is the solution of the time dependent Schrödinger equation for the initial state |*k*⟩. A simple example where the coefficients, ρ_*k*_, are easy to obtain is a thermal initial state. Then ρ_*k*_ = exp­(−β*E*_*k*_)/*Z*, where *Z* is the partition function that ensures normalization and β is the usual inverse temperature in units of Boltzmann’s constant. *E*_*k*_ is the *k*th eigenvalue of the Hamiltonian of the unperturbed oscillator. The **P**_*k*_(0)^′^ *s* are the projectors on the unperturbed states, **P**_*k*_(0) = **E**_*kk*_. Explicitly for the Morse oscillator **E**_*kk*_ = |*j*,*k*⟩⟨*j*,*k*| where *j* is fixed.

Another route to the form of the density matrix for a thermal initial state is

11 $$\rho \left(t\right)=U\left(t\right)\left(\frac{1}{Z}\exp\left(-\beta H_{0}\right)\right)U^{†}\left(t\right)=\frac{1}{Z}\exp\left(-\beta {U\left(t\right)H}_{0}U^{†}\left(t\right)\right)$$

This thereby shows that the **P**_*k*_(*t*)^′^ *s* are the eigen projectors of the Hamiltonian **U**(*t*)**H**_0_ **U** ^†^(*t*). More generally, whenever the initial density is a matrix of maximal entropy and it is propagated under a quantum unitary evolution operator, it remains of maximal entropy subject to constraints that are time dependent constants of the motion. Substituting the maximal entropy form of the density matrix (eqs 6 and 7) into **ρ**(*t*) = **U**(*t*)**ρ**(0)**U** ^†^(*t*) yields

$$\rho \left(t\right)=U\left(t\right)\left(\frac{1}{Z}\exp\left(-\sum_{k}\lambda_{k}\left(0\right)X_{k}\right)\right)U^{†}\left(t\right)=\frac{1}{Z}\exp\left(-\sum_{k}\lambda_{k}\left(0\right)\left(U\left(t\right)X_{k}U^{†}\left(t\right)\right)\right)$$

A simple limit where the results are clear is when the perturbation is slow enough to be adiabatic. Then the solution of the time dependent Schrödinger equation for the initial state |*k*⟩ is the adiabatic state |*k*_*a*_,*t*⟩, an eigenstate of the Hamiltonian at time *t* which can be determined by diagonalization of a *K* × *K* Hermitian matrix, **H**_0_ + **V**(*t*).

The Hamiltonian at time *t* is usually far simpler than **U**(*t*)**H**_0_ **U** ^†^(*t*). In the absence of level crossings, the state |*k*_a_,*t*⟩ is uniquely related to the unperturbed state |*k*⟩. Note that the coefficients *p*_*k*_ in eq ([Disp-formula eq9]) for **ρ**(*t*) remain time independent and equal to their initial value. For a thermal initial state one can write ρ_a_(*t*) = (1/*Z*)­exp­(−β∑_*k*_ *E*_*k*_ **P**_*k*a_(*t*)), where the projectors are defined **P**_*k*a_(*t*) ≡ |*k*_a_,*t*⟩⟨*k*_a_,*t*|. Note that the sum in the exponent is not the Hamiltonian matrix at time *t*. This is clear from a maximal entropy point of view. We are not given that the Hamiltonian at time *t* is a constant of the motion. What is a constant is **U**(*t*)**H**_0_ **U** ^†^(*t*). In the adiabatic approximation, where up to the Berry phase, **U**(*t*) shifts |*k*⟩ to |*k*_a_,*t*⟩. The constant of the motion is ∑_*k*_|*k*_a_,*t*⟩⟨*k*|**H**_0_|*k*⟩⟨*k*_a_,*t*| that equals the sum in the exponent, **H**_0_|*k*⟩ = *E*_*k*_|*k*⟩.

### Lossless Compaction in Practice

To implement the time dependent operators that are needed for a lossless, exact compaction one needs to determine **U**(*t*) explicitly. The method of Wei and Norman^64,65^ states that, if one has a basis {**X**_*i*_}, which is closed with itself and the Hamiltonian, i.e. **H**(*t*) = ∑_*k*_ *h*_*k*_(*t*)**X**_*i*_, one can write a unitary time evolution operator in the product form $U\left(t\right)=\prod_{k}\exp\left(g_{k}\left(t\right)X_{k}\right)$ . As any Hamiltonian matrix is linear in the set {**E**_*ik*_} and therefore closed, one can write the general form of the time evolution operator as

12 $$U\left(t\right)=\prod_{i,k}\exp\left(g_{i,k}\left(t\right)E_{i,k}\right)$$

Section S1 of the Supporting Information gives the derivation of the equations of motion of the group parameters of eq 12, the {*g*_*i*,*k*_(*t*)}.

The lossless, exact compaction requires one to diagonalize Hermitian matrices of dimension *K* by *K*. The number of vibrational bound states of a diatomic is seldom more than two or at most three dozen. Still, one needs to get also the higher eigenstates accurately. There is nothing “to show” because the lossless, exact compaction of the density matrix fully recovers uncompacted the density matrix.

### The Lossy Compaction

Generally speaking, if one does not use a full set of constraints when maximizing the entropy of the density matrix, the compaction will be lossy. In this case, Δ*S* will be positive because the information content of the constraints removed from the full set will not be in the density matrix constructed from the reduced set. This absent information will mean the entropy of the reconstructed density matrix is not maximally lowered.

It is interesting to comment on the special case of when the state is propagated under a unitary but not exact evolution operator **Ŭ**(*t*) that differs from the exact **U**(*t*). Where **ρ**(*t*) is the exact description of a system, evolved under the exact time evolution operator, **ρ**(*t*) = **U**(*t*)**ρ**(0)**U** ^†^(*t*), and **ρ̆** is the inexact description, evolved under the inexact time evolution operator, $\mathrm{\rho ̆}\left(t\right)=Ŭ\left(t\right)\rho \left(0\right){Ŭ}^{†}\left(t\right)$ , then

$$Tr\left(\rho \left(t\right)\ln\left(\mathrm{\rho ̆}\left(t\right)\right)\right)=Tr\left(U\left(t\right)\rho \left(0\right)U^{†}\left(t\right)Ŭ\left(t\right)\ln\left(\rho \left(0\right)\right){Ŭ}^{†}\left(t\right)\right)=Tr\left({Ŭ}^{†}\left(t\right)U\left(t\right)\rho \left(0\right)U^{†}\left(t\right)Ŭ\left(t\right)\ln\left(\rho \left(0\right)\right)\right)$$

There are many such options for **Ŭ**(t) which can compress the surprisal of the density matrix in a way not possible for **U**(*t*), for example the unitary evolution operator in the sudden approximation,^3^ or more generally when one fails to include one or more terms in the product form of the evolution operator. The operator ${Ŭ}^{†}\left(t\right)U\left(t\right)$ is unitary, but except at *t* = 0, it is not the identity matrix. It generates a state ${\mathrm{\rho ̆}\left(0\right)=Ŭ}^{†}\left(t\right)U\left(t\right)\rho \left(0\right)U^{†}\left(t\right)Ŭ\left(t\right)$ **ρ̆**(0) is a state that has been propagated from time *t* = 0 to *t*_f_ under the exact dynamics, **U**(*t*), and was then propagated backward in time from time *t* = *t*_f_ to 0 under the unitary but approximate dynamics, **Ŭ**(*t*). Because ${Ŭ}^{†}\left(t\right)U\left(t\right)\neq I$ , therefore **ρ̆**(0) ≠ ρ(0). Hence lossy compaction by a unitary-but-inexact operator **Ŭ**(t) is not reversible by the unitary-exact operator **U**(*t*). It is a different initial state, a state that gives exact propagation under the approximate unitary operator **Ŭ**(*t*): $Ŭ\left(t\right)\mathrm{\rho ̆}\left(0\right){Ŭ}^{†}\left(t\right)=U\left(t\right)\rho \left(0\right)U^{†}\left(t\right)$ .

Good compression, i.e. greatest compaction with minimal loss of information, generally takes advantage of the specific structure of the problem of interest. We begin with a very simple unitary propagation for the same problem. It is simple because we do not derive a general expression for an evolution operator, but only the results of a unitary propagation.

Recall the algebraic operators of the Hamiltonian of the perturbed oscillator, eq 1, i.e. **J** ^2^, **J**_*z*_^2^ and **J**_*x*_. These operators do not form a closed set: [**J**_*z*_^2^,**J**_*x*_] = *i*(**J**_*z*_ **J**_*y*_ + **J**_*y*_ **J**_*z*_), [**J**_*z*_^2^,**J**_*z*_ **J**_*y*_] = −*i*(**J**_*z*_^2^ **J**_*x*_ + **J**_*z*_ **J**_*x*_ **J**_*z*_), and on to higher order terms. However, they can still be used to write an inexact but unitary ansatz of the time evolution operator in the product form

13 $$Ŭ\left(t\right)=\exp\left(g_{1}\left(t\right)J^{2}\right)\exp\left(g_{2}\left(t\right){J_{z}}^{2}\right)\exp\left(g_{3}\left(t\right)J_{x}\right)$$

As the three operators **J** ^2^, **J**_*z*_^2^and**J**_*x*_ are Hermitian, the {*g*_*i*_(*t*)} need to be purely imaginary so that the factors are unitary. The explicit time dependence of the parameters {*g*_*i*_} is generally dropped henceforth.

We restrict our working to a specific molecule and therefore to the basis of a given *j* in which the operators have a finite representation, see eqs 3 to 5. As such, the action of **J** ^2^ is just a multiplication by a constant, **J** ^2^ = *j*(*j* + 1)**I**, and hence the factor exp­(*g*_1_ **J** ^2^) can be disregarded. In the unperturbed basis **J**_*z*_^2^ is diagonal, exp­(*g*_2_ **J**_*z*_^2^)|*j*,*m*⟩ = exp­(*g*_2_ *m* ^2^)|*j*,*m*⟩, while **J**_*x*_ connects the unperturbed state |*j*,*k*⟩ to its nearest neighbors, |*j*,*k* ± 1⟩. Therefore, one can write that $\exp\left(g_{3}J_{x}\right)|j,k\rangle =\sum_{m}c_{k,m}|j,m\rangle$ and $\exp\left(g_{2}{J_{z}}^{2}\right)\exp\left(g_{3}J_{x}\right)|j,k\rangle =\sum_{m}\exp\left(g_{2}m^{2}\right)c_{k,m}|j,m\rangle$ . Taking the initial state to be a stationary mixture, **ρ**(0) = ∑_*k*_ *p*_*k*_|*j*,*k*⟩⟨*j*,*k*|, then $\mathrm{\rho ̆}\left(t\right)=Ŭ\rho \left(0\right){Ŭ}^{†}$ can be calculated as

14 $$\exp\left(g_{2}{J_{z}}^{2}\right)\exp\left(g_{3}J_{x}\right)\rho \left(0\right)\exp\left(-g_{3}J_{x}\right)\exp\left(-g_{2}{J_{z}}^{2}\right)=\sum_{k}p_{k}\sum_{m}\sum_{n}{\exp\left(ig_{2}\left(n^{2}-m^{2}\right)\right)c}_{k,m}c_{k,n}^{*}|j,m\rangle \langle j,n|$$

This reduction in the size of the unitary time evolution operator, from eqs 12 to 13, is significant but of limited novelty. The populations are governed entirely by the term exp­(*g*_3_ **J**_*x*_) and, consequently, for calculating the population distributions, the time evolution operator, eq ([Disp-formula eq13]), is not an improvement on the sudden approximation time evolution operator **U** ^sud^(*t*) = exp­(−2*i*∫_0_^*t*^ *f*(*t* ^′^)­d*t* ^′^ **J**_*x*_).^3^

The populations, i.e. the terms where *m* = *n* in eq 14, are the $\sum_{k}p_{k}\sum_{m}{\left|c_{k,m}\right|}^{2}|m\rangle \langle m|$ . With regards to the coherences, eq 13 is an improvement on **U** ^sud^(*t*). **U** ^sud^(*t*) calculates finite absolute values of the coherences. In terms of eq 14, their magnitude is the reasonable value $\sqrt{{\left|c_{k,m}\right|}^{2}{\left|c_{k,n}\right|}^{2}}$ . Equation 13 gives them improved oscillatory behavior, with the expected frequencies, as the factor exp­(*g*_2_(*t*)**J**_*z*_^2^) contains the information about the energy differences of the molecular states. However, phase differences are still present in the coherences.

We will regard the approximation above as a first step in a sequence of unitary but approximate Wei-Norman like product expressions. They are all approximate because an exact Wei-Norman factorization requires a closed Lie algebra. Recall however our key point, when working in a matrix representation of the operators, there always exists a Lie algebra, namely the Gelfand matrices, with which the operators are closed under commutation. Any matrix can be written as a linear sum of the set of Gelfand matrices. It is very useful to work with the *K* ^2^ matrices **E**_*nm*_, eq 12, but it is strictly not necessary. This is because, by the Cayley-Hamilton theorem^66^ (see also ref 67), a matrix that represents an angular momentum operator power *K* or higher can be expressed as linear combination of the same matrix at lower powers. Thereby, the infinite series which results on applying the Wei-Norman factorization can be made finite. In practice we have already shown how to do it^3^ using the Sylvester formula^68–70^ for a power of a matrix.

### Lossy Compaction in Practice: An Example of How to Compact with Minimal Loss of Information

This section will demonstrate the lossy compaction of the surprisal of the dynamical system of an anharmonic Morse oscillator perturbed by an external force.

The density matrix in Schrödinger form, eq 11, is **ρ**(*t*) = (1/*Z*) ***U*** exp­(−β**H**_0_)**U** ^†^ where *Z* is the time independent partition function such that 1/Z≡exp(ZI) . Using eq 1 in eq 11,

$$\rho \left(t\right)=\left(1/Z\right)U\left(t\right)\exp\left(-\beta A\left(J^{2}-{J_{z}}^{2}\right)\right){U\left(t\right)}^{†}=\exp\left(ZI-\beta AU\left(t\right)\left(J^{2}-{J_{z}}^{2}\right){U\left(t\right)}^{†}\right)$$

**I** is the constraint which ensures normalization, with a time independent Lagrange multiplier, Z . By the equality of eqs 11 and 6, we get

15 $$ZI-\beta AJ^{2}+\beta AU\left(t\right){J_{z}}^{2}{U\left(t\right)}^{†}=\sum_{k}\lambda_{k}\left(t\right)X_{k}$$

Using the Campbell lemma, exp­(**A**)**B** exp­(−**A**) = exp­([**A**, ])**B**, and the Gelfand forms of ${J_{z}}^{2}=\sum_{m_{j}=-j}^{j}{m_{j}}^{2}E_{m_{j},m_{j}}$ , and equation 12, one can write

16 $$U{J_{z}}^{2}U^{†}=\sum_{m_{j}=-j}^{j}{m_{j}}^{2}\left(\prod_{i,k=-j}\exp\left(g_{i,k}\left[E_{i,k},\right]\right)\right)E_{m_{j},m_{j}}$$

Inserting eq 16 into eq 15, along with the Gelfand basis forms of **J** ^2^ = *j*(*j* + 1)**I** and $I=\sum_{m_{j}=-j}^{j}E_{m_{j},m_{j}}$ , yields

17 $$\left(Z-\beta Aj\left(j+1\right)\right)\sum_{m_{j}=-j}^{j}E_{m_{j},m_{j}}+\beta A\sum_{m_{j}=-j}^{j}{m_{j}}^{2}\left(\prod_{i,k=-j}^{j}\exp\left(g_{i,k}\left[E_{i,k},\right]\right)\right)E_{m_{j},m_{j}}=\sum_{k}\lambda_{k}\left(t\right)X_{k}$$

Through a series of manipulations described in detail in Section 2 of the Supporting Information, the LHS of eq 17 can be written as a sum over Gelfand matrices with coefficients which are functions of the time dependent group parameters of eq 12

18 $$\sum_{i,k}\phi_{i,k}\left(\{g_{m,n}\}\right)E_{i,k}=\sum_{k}\lambda_{k}\left(\{g_{m,n}\}\right)X_{k}$$

These group parameters, in terms of which the ϕ_*i*,*k*_({*g*_*m*,*n*_}) and hence the λ_*k*_({*g*_*m*,*n*_}) are defined, are calculated using the method of Wei and Norman^64,65^ explained in detail in.^3,62,71,72^ A brief description of this method is also given in the Supporting Information, Section 1. Henceforth, for simplification, objects which are a function of the group parameters will be written as, for example, λ_*k*_(*g*) ≡ λ_*k*_({*g*_*m*,*n*_}).

The RHS of eq ([Disp-formula eq18]) remains the surprisal of a density matrix of maximum entropy. The constraints of this surprisal, {**X**_*k*_}, have not yet been defined. Compaction is achieved by using a set of constraints which contain the structure of the angular momentum operators constituting the Hamiltonian: **J** ^2^, **J**_*z*_^2^ and **J**_*x*_. To derive such a set of constraints, it is advantageous to look at the system—a force perturbing an anharmonic Morse oscillator—in the Interaction Picture.

The Schrödinger Hamiltonian is **H**_*S*_ = **H**_0,*S*_ + **H**_1,*S*_(*t*), where **H**_0,*S*_ is eq 1 is and **H**_1,*S*_(*t*) is eq 2. The Interaction Picture Hamiltonian, **H**_*I*_ = **H**_0,*I*_ + **H**_1,*I*_(*t*) is

$$H_{I}=\exp\left(iH_{0,S}t\right)H_{S}\exp\left(-iH_{0,S}t\right)=H_{0,S}+\exp\left(iH_{0,S}t\right)H_{1,S}\left(t\right)\exp\left(-iH_{0,S}t\right)$$

with *ℏ* ≡ 1. The time independent part of the interaction Hamiltonian is just **H**_0,*I*_ = **H**_0,*S*_, and the time dependent part is

$$H_{1,I}\left(t\right)=2f\left(t\right)\exp\left(-iA{J_{z}}^{2}t\right)J_{x}\exp\left(iA{J_{z}}^{2}t\right)$$

**H**_1,*I*_(*t*) can be reformulated using the Campbell Lemma ([Proposition 3.35] of ref 2) and expanded as a Taylor series to yield a sum

19 $$\exp\left(-iAt\left[{J_{z}}^{2},\right]\right)J_{x}=\sum_{k=0}^{\infty }\alpha_{k}\left(t\right){\left[{J_{z}}^{2},\right]}^{k}J_{x}$$

In accordance with the Cayley-Hamilton theorem, because all of the parts of eq 19 are finite-dimensional matrices, the matrices in the sum on the RHS are only linearly dependent up to a finite *k*. As is discussed below, all of the {[**J**_*z*_^2^,]^*k*^ **J**_*x*_} are sub/super-diagonal matrices, meaning that there are only 2*j*-2 nonzero elements in the matrices on the LHS and RHS of eq 19. This means, that ([**J**_*z*_^2^,]^2*j*−2^ **J**_*x*_) = ∑_*k*=0_^2*j*–3^ *c*_*k*_([**J**_*z*_^2^,]^*k*^ **J**_*x*_) and, therefore, the RHS of eq 19 is a finite sum,

$$\exp\left(-iAt\left[{J_{z}}^{2},\right]\right)J_{x}=\sum_{k=0}^{2j-3}\beta_{k}\left(t\right){\left[{J_{z}}^{2},\right]}^{k}J_{x}$$

Therefore, $H_{I}=\sum_{k}h_{k,I}\left(t\right)X_{k}$ , where {**X**_*k*_} = {**J** ^2^,**J**_*z*_^2^,**J**_*x*_,[**J**_*z*_^2^,**J**_*x*_],([**J**_*z*_^2^,]^2^ **J**_*x*_),...,([**J**_*z*_^2^,]^2*j*−3^ **J**_*x*_)}. The set of matrices, {**X**_*k*_}, is constituted of three subsets:

1) A normalization constraint with a diagonal matrix structure
20 $$X^{N}=J^{2}=j\left(j+1\right)I$$
2) Constraints which distribute the population and have a diagonal matrix structure. Note, this subset has to be augmented with additional **J**_*z*_^2*n*^ terms to ensure the proper distribution of the populations
21 $$X^{D}=\left\{{J_{z}}^{2},{J_{z}}^{4},{J_{z}}^{6},...,{J_{z}}^{2j}\right\}=\left(\begin{array}{lllll} ■ & 0 & 0 & 0 & \ddots \\ 0 & ■ & 0 & \ddots & 0\\ 0 & 0 & \ddots & 0 & 0\\ 0 & \ddots & 0 & ■ & 0\\ \ddots & 0 & 0 & 0 & ■ \end{array}\right)$$
3) Constraints which move the populations between nearest neighbor states and have a sub- and super-diagonal matrix structure
22 $$X^{SD}=\left\{J_{x},\left[{J_{z}}^{2},J_{x}\right],\left({\left[{J_{z}}^{2},\right]}^{2}J_{x}\right),...,\left({\left[{J_{z}}^{2},\right]}^{2j-3}J_{x}\right)\right\}=\left(\begin{array}{lllll} 0 & ■ & 0 & 0 & \ddots \\ ■ & 0 & ■ & \ddots & 0\\ 0 & ■ & \ddots & ■ & 0\\ 0 & \ddots & ■ & 0 & ■\\ \ddots & 0 & 0 & ■ & 0 \end{array}\right)$$

Equations 20 to 22 collect into the set {**X**_*i*_} = {**J** ^2^,**J**_*z*_^2^,**J**_*z*_^4^···**J**_*z*_^2*j*^,**J**_*x*_,[**J**_*z*_^2^,**J**_*x*_]···[**J**_*z*_^2^,]^2*j*−3^ **J**_*x*_} = {**X** ^*N*^,**X** ^*D*^,**X** ^*SD*^}. Before this set can be used as the constraints for the surprisal, however, it requires orthonormalization using the Gram-Schmidt process. Using the Hilbert-Schmidt inner product of two matrices as (**X**_*i*_|**X**_*k*_) ≡ Tr­(**X**_*i*_^†^·**X**_*k*_),^5^ the basis is orthogonalized as

$${\mathrm{X̃}}_{i}=X_{i}-\sum_{k=1}^{i-1}{\mathrm{X̃}}_{k}\frac{\left({\mathrm{X̃}}_{k}|X_{i}\right)}{\left({\mathrm{X̃}}_{k}|{\mathrm{X̃}}_{k}\right)}$$

and then normalized as

$${\mathrm{X̌}}_{i}={\mathrm{X̃}}_{k}/\sqrt{\left({\mathrm{X̃}}_{k}|{\mathrm{X̃}}_{k}\right)}$$

This procedure produces an orthonormal basis constituted of a normalization operator, a diagonal subset and a super/sub-diagonal subset, in which the surprisal can be constructed.

23 $$\left\{{\mathrm{X̌}}_{i}\right\}=\left\{{\mathrm{X̌}}^{N},{\mathrm{X̌}}^{D},{\mathrm{X̌}}^{SD}\right\}=\left\{{\mathrm{X̌}}_{0},{\mathrm{X̌}}_{1}^{D},{\mathrm{X̌}}_{2}^{D},{\mathrm{X̌}}_{3}^{D}\cdot \cdot \cdot ,{\mathrm{X̌}}_{j}^{D},{\mathrm{X̌}}_{1}^{SD},{\mathrm{X̌}}_{2}^{SD},{\mathrm{X̌}}_{3}^{SD}\cdot \cdot \cdot {\mathrm{X̌}}_{2j-3}^{SD}\right\}$$

when this set is used on the RHS of eq 24

24 $$\sum_{i,k}\phi_{i,k}\left(g\right)E_{i,k}\overset{compaction}{\Rightarrow }\lambda_{0}\left(g\right){\mathrm{X̌}}_{0}+\sum_{k=1}^{j}\lambda_{k}^{D}\left(g\right){\mathrm{X̌}}_{k}^{D}+\sum_{k=1}^{2j-2}\lambda_{k}^{SD}\left(g\right){\mathrm{X̌}}_{k}^{SD}$$

the O(j) number of $\left\{{\mathrm{X̌}}_{i}\right\}$ will be significantly less than the $O\left(j^{2}\right)$ number of **E**_*i*,*k*_^′^ *s* on the LHS. This action, going from eq 18 to 24, compresses the surprisal. This compaction is lossy. The LHS of eq 24 contains all of the information about the dynamical system. Some of this information is lost, however, when we use the RHS of eq 24 as the compressed surprisal.

Due to the symmetry, it is the case that ϕ_*k*,*k*+1_(*g*) = ϕ_*k*+1,*k*_(*g*), therefore

25 $$\sum_{k}\phi_{k,k+1}\left(g\right)\left(E_{k,k+1}+E_{k+1,k}\right)=\sum_{k=1}^{2j-2}\lambda_{k}^{SD}\left(g\right){\mathrm{X̌}}_{k}^{SD}$$

Also

26 $$\sum_{k}\phi_{k,k}\left(g\right)E_{k,k}=\lambda_{0}\left(g\right){\mathrm{X̌}}_{0}+\sum_{k=1}^{j}\lambda_{k}^{D}\left(g\right){\mathrm{X̌}}_{k}^{D}$$

The RHS of eq 24 retains the information in eqs 25 and 26. Equation 24) is a lossy compression, however, because there is some information in the LHS which is not in the RHS. The constraints which contain the information which is lost in the compression are the off-diagonal Gelfands beyond the sub- and super diagonal lines, the **E**_*i*,*k*_ for abs­(*i* – *k*) > 1

27 $$\sum_{i\neq k,i\neq k\pm 1}\phi_{i,k}\left(g\right)E_{i,k}=\left(\begin{array}{llllll} 0 & 0 & ■ & ■ & ■ & \ddots \\ 0 & 0 & 0 & ■ & \ddots & ■\\ ■ & 0 & 0 & \ddots & ■ & ■\\ ■ & ■ & \ddots & 0 & 0 & ■\\ ■ & \ddots & ■ & 0 & 0 & 0\\ \ddots & ■ & ■ & ■ & 0 & 0 \end{array}\right)$$

These constraints are removed in the compression of eq 24, but, as will be shown, this does not constitute a significant loss of information (see Figure 1). Combining eqs 25 and 26

28 $$\sum_{k}^{j}\left(\phi_{k,k}\left(g\right)E_{k,k}+\phi_{k,k+1}\left(g\right)\left(E_{k,k+1}+E_{k+1,k}\right)\right)=\lambda_{0}{\mathrm{X̌}}_{0}+\sum_{p=1}^{j}\lambda_{p}^{D}\left(g\right){\mathrm{X̌}}_{p}^{D}+\sum_{q=1}^{2j}\lambda_{q}^{SD}\left(g\right){\mathrm{X̌}}_{q}^{SD}$$

**Figure 1:**
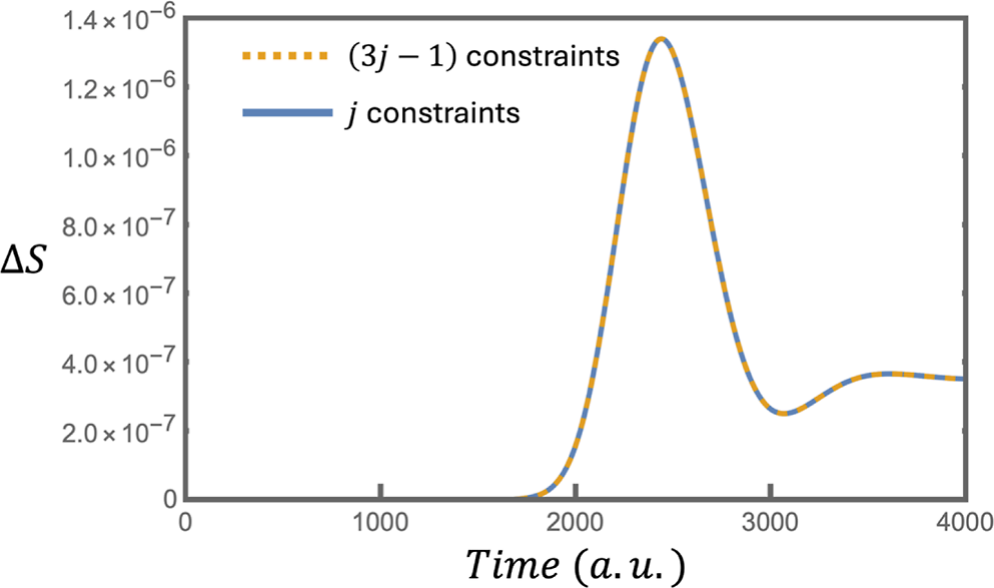
Comparison of the entropy difference, Δ*S*, between the exact density matrix calculated via the propagation of the Liouville von-Neuman equation and density matrices of maximal entropy constructed from 3*j* – 1 (yellow dashed line) and *j* (blue line) constraint in the surprisal.

This equation is an equality. On the LHS, the {ϕ_*k*,*k*_} are purely imaginary and {ϕ_*k*,*k*+1_} are complex. On the RHS, λ_0_ is time independent and the {λ_*p*_^D^} and {λ_*q*_^SD^}­are purely real. This means that both sides of eq 28 have approximately 3*j* time dependent parameters, and are therefore equally compact.

The dominant constraints on the RHS of eq 28 are those specified in eq 23 with lower value indices, ${\mathrm{X̌}}_{p}^{x}>{\mathrm{X̌}}_{p+1}^{x}$ where *x* = D or SD. Further compression occurs when one removes the subdominant constraints, ${\mathrm{X̌}}_{j}^{D},{\mathrm{X̌}}_{j-1}^{D}$ etc. and ${\mathrm{X̌}}_{2j}^{SD},{\mathrm{X̌}}_{2j-1}^{SD}$ etc.

29 $$\sum_{k}^{j}\left(\phi_{k,k}\left(g\right)E_{k,k}+\phi_{k,k+1}\left(g\right)\left(E_{k,k+1}+E_{k+1,k}\right)\right)\overset{compaction}{\Rightarrow }\lambda_{0}{\mathrm{X̌}}_{0}+\sum_{p=1}^{\#<j}\lambda_{p}^{D}\left(g\right){\mathrm{X̌}}_{p}^{D}+\sum_{q=1}^{\#<2j}\lambda_{q}^{SD}\left(g\right){\mathrm{X̌}}_{q}^{SD}$$

As will be shown, the information lost by the removal of a significant number of subdominant constraints is negligible in systems for which the model of the perturbed anharmonic oscillator used in this work is valid (Figure 1).

Overall, the two compressions developed in this section (eqs 24 and 29) have reduced the number of constraints in the surprisal from $O\left(j^{2}\right)$ down to O(j) .

### Numerical Example to Quantify the Information Lost in the Compression

To understand what information is lost in the two compactions of eqs 24 and 29, a numerical example will be given. A density matrix of maximum entropy is produced by the compression of a density matrix calculated with the Liouville-von Neumann equation

30 $$i\frac{\partial \rho^{LvN}}{\partial t}=\left[H,\rho^{LvN}\right]$$

The Hamiltonian **H**(*t*) = **H**_0_ + **V**(*t*) comes from eqs 1 and 2. The perturbation caused to the system is the time-dependent force, *f*(*t*), of a structureless particle moving along a classical trajectory and perturbing the oscillator along the *x*-axis,

31 $$f\left(t\right)=\frac{f}{\cosh\left(\left(t-t_{0}\right)/\tau \right)}$$

where *t*_0_ is the center of the perturbation, it is time of greatest magnitude, *f* is the scale factor of the perturbation and τ is its duration. The perturbation parameters in eq 31 are *f* = 9 × 10^–5^ a.u., τ = 240 a.u. and *t*_0_ = 2000 a.u. Figure S1 in Section 3 of the Supporting Information shows this *f*(*t*).

The anharmonic oscillator is a 33-state molecule (*j* = 32) with parameter *A* = 20 cm^–1^ in eq 1. The temperature of the system is *T* = 1000 K, therefore giving a value of β = 315.6 a.u., which defines the initial unperturbed thermal equilibrium population distribution.

The initial entropy of the anharmonic oscillator in thermal equilibrium at *T* = 1000 K is *S*_0_ = −*tr*[**ρ**_0_ ln­(ρ_0_)] = 1.235. This value is to be compared with the entropy when the populations are distributed uniformly over all the bounds states, i.e. ρ_*m*,__*m*_^uni^ = 1/(2*j* + 1) ∀*m*. The uniform distribution entropy is *S* ^uni^ = −*tr*[**ρ** ^uni^ln­(**ρ** ^uni^)] = 4.174. *S* ^uni^ quantifies the maximum uncertainty, or minimum information, one could possibly have about the bound state system. The lower value of 1.235 reflects the information one has by virtue of one’s knowledge that ρ_0_ is in a thermal equilibrium state. This accounts for the entropy difference, Δ*S* = *S* ^uni^ – *S*_0_ = 2.939, which quantifies this additional information.

From eqs 6 and 7, the density matrix of maximal entropy is $\rho^{ME}\equiv \exp\left(-\sum_{k}\lambda_{k}\left(t\right){\mathrm{X̌}}_{k}\right)$ . The constraints in the surprisal are the orthonormal set of eq 23. To understand the information lost in the compression of **ρ** ^*LvN*^, the time dependent Lagrange parameters {λ_*k*_(*t*)} are calculated by taking the inner product of the constraints with the density matrix,

$$\lambda_{k}\left(t\right)=\left({\mathrm{X̌}}_{k}|\rho^{LvN}\left(t\right)\right)$$

Equation 8 provides a measure of the increase in uncertainty, or loss of information, resultant from the compaction, Δ*S* = Tr­[**ρ** ^LvN^·ln­(**ρ** ^LvN^/**ρ** ^ME^)].

Figure 1 provides a quantification of the information lost by the compactions of eqs 24 and 29. The orange dashed line in Figure 1 shows the Δ*S* when **ρ** ^ME^ is calculated using the 3*j* – 2 constraints in eqs 21 and 22, with the normalization constraint in eq 20, thereby fully accounting for all of the diagonal, subdiagonal and superdiagonal elements in the surprisal. The orange dashed line therefore quantifies the uncertainty caused by the compaction of the surprisal in eq 24. Another way of putting this is to say that this line quantifies the information contained in eq 27 which is not included in the surprisal. After the perturbation is over, Δ*S* is approximately 7 orders of magnitude lower than *S*.

The blue line in Figure 1 shows the Δ*S* when **ρ** ^ME^ is calculated using only the *j* dominant constraints. This is the compaction which occurs when one removes the subdominant constraints of $\left\{{\mathrm{X̌}}^{D}\right\}$ and $\left\{{\mathrm{X̌}}^{SD}\right\}$ from the surprisal. The *j* constraints retained in the surprisal are the normalization constraint, the first eight dominant constraints from $\left\{{\mathrm{X̌}}^{D}\right\}$ , and the first 24 dominant constraints from $\left\{{\mathrm{X̌}}^{SD}\right\}$ The convergence of the two lines in Figure 1 shows that the information contained in the 2*j* subdominant constraints which are discarded in eq 29 is negligible.

Figure S2 in Section 4 of the Supporting Information shows the fidelities of the two compacted **ρ** ^ME^, *F*(**ρ** ^LvN^,**ρ** ^ME^). The two validation measures show the same structure. The loss of fidelity from perfect *F* = 1, Δ*F* = 1 – *F*, is approximately proportional to the entropy difference at all times,

$$\Delta F\left(\rho^{LvN}\left(t\right),\rho^{ME}\left(t\right)\right)\propto ̃\Delta S\left(t\right),\forall t$$

On the fidelity scale of 0 to 1, where here Δ*F* = 0 means no loss of information, max­(Δ*F*) < 2 × 10^–7^. By both validity measures, therefore, the information lost when one removes the off-diagonal elements of the surprisal and the 2*j* subdominant constraints from $\left\{{\mathrm{X̌}}^{D}\right\}$ and $\left\{{\mathrm{X̌}}^{SD}\right\}$ is negligible. In other words, removing these $O\left(j^{2}\right)$ constraints does not significantly increase the uncertainty about the system.

Figures 2 and 3 compare the elements of **ρ** ^LvN^ and the **ρ** ^ME^ constructed with *j* constraints in the surprisal. Figures 2 and 3 compare the ground state populations and the real part of the coherence between the ground and the first excited state, respectively. Section 5 of the Supporting Information provides an elementwise comparison of additional populations and coherences in **ρ** ^LvN^ and this **ρ** ^ME^.

**Figure 2:**
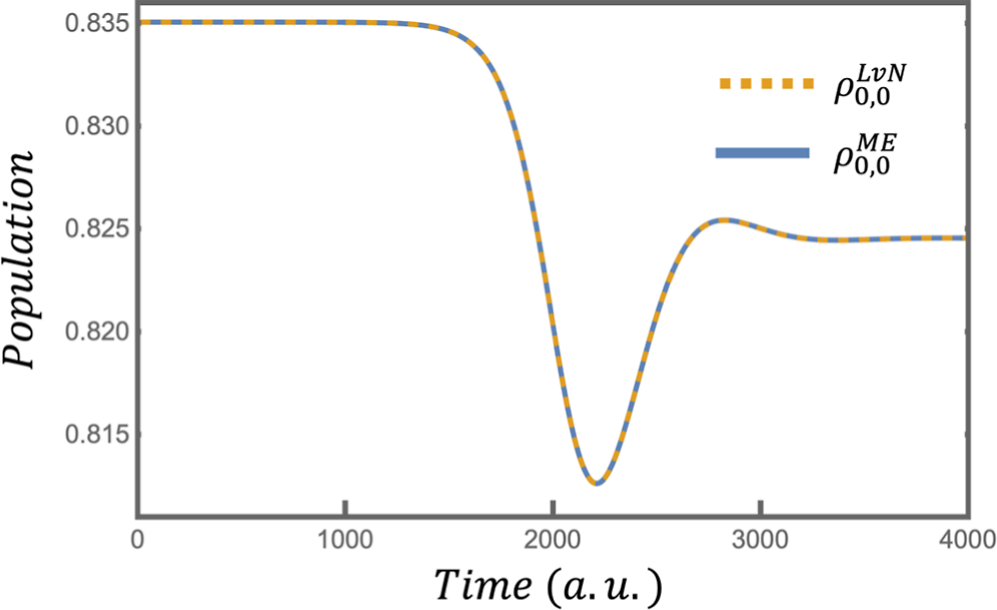
Comparison of the ground state populations of the exact ρ^LvN^ (yellow dashed line) and the compacted ρ^ME^ (blue line) when the latter is constructed with *j* constraints in the surprisal.

**Figure 3:**
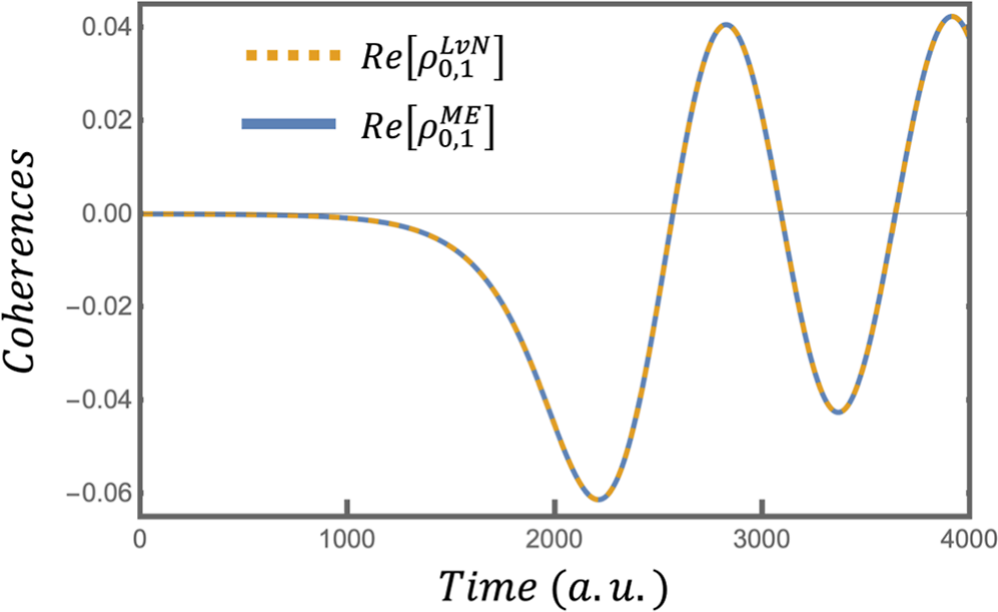
Comparison of the real part of the coherence between the ground and first excited states in the exact ρ^LvN^ (yellow dashed line) and the compacted ρ^ME^ (blue line) when the latter is constructed with *j* constraints in the surprisal.

The perturbation depopulates the ground state by ρ_0,0_^LvN^(*t*_f_)/ρ_0,0_^LvN^(0) = 0.987, augments the population of the first excited state by ρ_1,1_^LvN^(*t*_f_)/ρ_1,1_^LvN^(0) = 1.049, ρ_2,2_^LvN^(*t*_f_)/ρ_2,2_^LvN^(0) = 1.116 etc.. If one used a stronger perturbation, i.e. a greater *f*, Δ*S* would increase because the perturbation would increase the strength of the coherences between states with larger Δ*m*_*j*_. In other words, the constraints in eq 27 would become more significant. On the other hand, if one were to use a molecule with more bound states, i.e. with a larger value of *j*, Δ*S* would decrease because the additional constraints in eq 27 for more, higher Δ*m*_*j*_ coherences, would contain far less information than the additional constraints in eqs 25 and 26.

Because the model for the system we are using does not include continuum states, we are limited in the strength of the perturbation we can use. Our model would become less and less valid is we used perturbations which more and more populated the highest bound states. Therefore, the model of the anharmonic oscillator we are using limits us to perturbations with a scaling factor of around *f* = 9 × 10^–5^ a.u.

This section has shown that the surprisal of this dynamical system (up to about 2000 a.u. after the maximum of the perturbation, the point at which the perturbation is “over”—see Figure S1 of the Supporting Informartion) can be compacted from (2*j*+1)^2^ constraints down to *j* with a minimal loss of information. To quantify this statement, the information lost in the compaction, Δ*S*, is approximately 7 orders of magnitude lower than the information in the uncompacted system, *S*. This meets the specifications for good compression given above: significant compaction ( $O\left(j^{2}\right)$ constraints down to *j*) with minimal loss of information.

This section has demonstrated the compression of the surprisal, by deriving the Lagrange parameters from a density matrix calculated using the Liouville von-Neuman equation. It must be emphasized, however, that prior calculation of **ρ** ^LvN^ is not required to calculate **ρ** ^ME^. As eq 18 shows, the Lagrange parameters are functions of the group parameters of the unitary time evolution operator, eq 12, which can be calculated using the Wei-Norman method as described in Section 1 of the Supporting Information. Analytic expressions of the Lagrange parameters of a minimal, *j* = 1 system, are shown in Section 6 of the Supporting Information.

### Perspectives

We have shown how to compact the dynamics of a perturbed anharmonic system by working with the energy eigenstates of the unperturbed system. An anharmonic molecule has a finite number *K* of bound states. *K* variables that are functions of state, and therefore functions of time, can exactly represent any Hermitian density matrix. If all *K* are linearly independent, the entropy of the state is globally maximal, and no real compaction is possible. Under an exact unitary time evolution, the entropy of the state is conserved in time. Therefore, lossless compaction is possible when the initial state is not fully random and so its entropy is maximal but below its global limit. For such an initial state we show how to compute the relevant *K* variables, and also approximations thereof that provide a lossy compression. The operators, the expectation values of which constitute these same variables, when used as constraints in a maximum entropy formalism provide an exact time dependent density matrix. Using fewer constraints determines an approximate density matrix of a higher entropy.

We show a constructive approach where we can compute the *K* variables from first principles. One can also use the approach to compact an already given density matrix. It is also possible to provide an approximate lossy compaction. This is typically what is done in surprisal analysis when the leading variables are referred to as the dominant constraints.^73^

An alternative to working in the energy representation is to work in the time domain. There we showed^51^ that one can exactly compact the time dependence given the exact density matrix at a sufficient number of time points. This has the advantage that it inherently orders the variables in order of dominance. On the other hand, it determines the constraints numerically. It remains an open task to determine the constraints analytically.

We have discussed the dynamics in the subspace of bound states. There are Lie algebras for the continuum part of anharmonic potentials, for example.^9,74^ The analog of the discrete vibrational quantum number is continuous. So one needs to rephrase the present approach. Even more so one needs a unified approach that can describe perturbations that induce transitions from bound states to the continuum.^75^

Entropy and maximum entropy, constraints, variables of state, conservation laws that morph to slow varying variables etcetera, have all originated in discussions of open macroscopic systems. Our discussion uses in an essential way that the system is closed. Lie algebraic methods have been applied in open nonlinear systems, for example ref 76. For a closed system we could apply maximal entropy, and we could add constraints all the way to exact reproduction of the dynamics. How far we can proceed for open systems is a question for further research.

## Supplementary Material



## Footnotes

a The algebraic quantum number goes from −*j* to *j*.

## Notes

SI_JCTC_Submission_ACSFormatted.pdf which contains six sections. Section 1: the derivation of the equations of motion of the group parameters of the factorized time evolution operator using the method of Wei and Norman Section 2: explanation of algorithms used in the derivation of eq 30 of the main text and elsewhere Section 3: the perturbation used in the numerical results section of this work Section 4: she fidelities of the reconstructed density matrices of maximal entropy, with once and twice compacted surprisals, to the uncompressed exact density matrix Section 5: an extended elementwise comparison of an exactly time-propagated density matrix with a density matrix of maximum entropy that has a compacted surprisal. Section 6: an explicit analytical derivation of the Lagrange parameters of a system with a small *j* = 1 molecule (PDF)
