# Supplementary material for: Lossless and Lossy Characterization of the State of Perturbed Anharmonic Diatomics: An Information-Theoretic Compaction of Quantum Dynamics
Source: J Chem Theory Comput. 2026 Feb 18;22(5):2194–205. doi: 10.1021/acs.jctc.6c00025 (PMC12980743; doi:10.1021/acs.jctc.6c00025)
Supplement: Supplementary file 1 [file ct6c00025_si_001.pdf]

Supplementary Information: Lossless and lossy characterization of the state of perturbed anharmonic diatomics: an information-theoretic compaction of quantum dynamics

James R. Hamilton[1] *and Raphael D. Levine*[1,2,*]

[1]The Fritz Haber Center for Molecular Dynamics, Institute of Chemistry, The Hebrew University of Jerusalem; Jerusalem 91904, Israel.

[2]Department of Molecular and Medical Pharmacology, David Geffen School of Medicine and Department of Chemistry and Biochemistry, University of California; Los Angeles, CA 90095, United States.

[*]Author to whom correspondence should be addressed: raphy@mail.huji.ac.il

Section 1: The derivation of the equations of motion of the group parameters of the factorised time evolution operator using the method of Wei and Norman

The time evolution operator $\mathbf{U}(t)$ can be written in a product form of time independent operators, $\{\mathbf{Y}_k\}$, and time dependent group parameters, $\{g_i(t)\}$, à la Wei and Norman[1, 2] as discussed in [3-5] and [6].

$$\mathbf{U}(t) = \prod_i \exp(g_i(t)\mathbf{Y}_k) \quad (S1)$$

This form of the time evolution operator has the requirement that the operators $\{\mathbf{Y}_k\}$ form a closed set under commutation

$$[\mathbf{Y}_m, \mathbf{Y}_n] = \sum_k c^k_{m,n}\mathbf{Y}_k \quad (S2)$$

Where $\{c^k_{m,n}\}$ are the structure constants. Further, the $\{\mathbf{Y}_i\}$ must be closed with the Hamiltonian

$$[\mathbf{H}(t), \mathbf{Y}_n] = \sum_k b^k_n(t)\mathbf{Y}_k \quad (S3)$$

This condition is fulfilled when the Hamiltonian can be written as a sum over the basis

$$\mathbf{H}(t) = \sum_k h_k(t)\mathbf{Y}_k \quad (S4)$$

One suitable $\{\mathbf{Y}_k\}$, which is closed with any bound Hamiltonian, is the Gelfand basis $\{\mathbf{E}_{i,k}\}$. Any finite dimensional Hamiltonian can be written as a sum over the Gelfand Operators $\mathbf{H}(t) = \sum_k h_{i,k}(t)\mathbf{E}_{i,k}$, and with it equation (S1) becomes equation (12) of the main text, $\mathbf{U}(t) = \prod_{i,k} \exp\left(g_{i,k}(t)\mathbf{E}_{i,k}\right)$.

Equation (S1) is differentiated with respect to time yielding

$$\frac{d\mathbf{U}}{dt} = \sum_{k=1}^{N} \frac{dg_k}{dt}\left(\prod_{m=1}^{k-1} \exp(g_m\mathbf{Y}_m)\right)\mathbf{Y}_k\left(\prod_{m=k}^{N} \exp(g_m\mathbf{Y}_m)\right) (S5)$$

Substituting the equation of motion of the time evolution operator, $d\mathbf{U}/dt = -i\mathbf{H}\mathbf{U}$, into the LHS of equation (S5), and multiplying from the right by $\mathbf{U}^{-1} = \prod_{m=N}^{1} \exp(-g_m\mathbf{Y}_m)$ yields

$$-i\mathbf{H} = \sum_{k=1}^{N} \frac{dg_k}{dt} \left(\prod_{m=1}^{k-1} \exp(g_m \mathbf{Y}_m) \right) \mathbf{Y}_k \left(\prod_{m=k-1}^{1} \exp(-g_m \mathbf{Y}_m) \right) (S6)$$

Using the Campbell lemma, $\exp(\mathbf{A})\mathbf{B}\exp(-\mathbf{A}) = \exp\left([\mathbf{A},\ ]\right)\mathbf{B}$ ([Proposition 3.35] of ref [7]) and equation (S4) for the form of the Hamiltonian, this equation becomes

$$\sum_{k} h_k(t)\mathbf{Y}_k = i \sum_{k=1}^{N} \frac{dg_k}{dt} \left(\prod_{m=1}^{k-1} \exp\left(g_m [\mathbf{Y}_m,\ ]\right) \right) \mathbf{Y}_k (S7)$$

Using the formulas discussed in section S2 of this SI, specifically equation (S15), this can be written as a system of coupled differential equations

$$\sum_{k} h_k(t)\mathbf{Y}_k = i \sum_{k=1}^{N} \frac{dg_k}{dt} \sum_{m=1}^{N} \xi_{m,k}\left(\{g_m\}\right) \mathbf{Y}_m \ (S8)$$

This can be written as a matrix equation in the standard operator basis in which the $\{\mathbf{Y}_m\}$ are represented as vectors of length N such that $\mathbf{Y}_1{}^T = (1 \quad 0 \quad 0 \quad ...)$, $\mathbf{Y}_2{}^T = (0 \quad 1 \quad 0 \quad ...)$ etc… In this basis, the vectors are defined $\mathbf{h}^T \equiv (h_1(t) \quad h_2(t) \quad h_3(t) \quad ...)$ and $\dot{\boldsymbol{g}}^T \equiv (dg_1/dt \quad dg_2/dt \quad dg_3/dt \quad ...)$. In addition, $\boldsymbol{\Xi}$ is defined as an $N \times N$ matrix with elements $\xi_{m,k}\left(\{g_m\}\right)$. Equation (S8) is hence written as the following matrix equation

$$\mathbf{h} = i\Xi(\boldsymbol{g})\,\dot{\boldsymbol{g}} (S9)$$

Or, alternatively,

$$\dot{\boldsymbol{g}} = -i\Xi(\boldsymbol{g})^{-1}\mathbf{h}\ (S10)$$

Which can be solved with standard numerical methods to find the group parameters $\{g_m\}$ as a function of time.

Section 2: Explanation of algorithms used in the derivation of equation (18) of the main text and elsewhere

This section derives equation (18) of the main text from equation (17)

$$\left(\mathcal{Z}-\beta \mathrm{A} j(j+1)\right)\sum_{m_j=-j}^{j}\mathbf{E}_{m_j,m_j}+\beta \mathrm{A}\sum_{m_j=-j}^{j} {m_j}^2\left(\prod_{i,k=-j}^{j}\exp\left(g_{i,k}[\mathbf{E}_{i,k},]\right)\right)\mathbf{E}_{m_j,m_j}=\sum_{k}\lambda_k(t)\,\mathbf{X}_k \quad (S11)$$

This equation is solved by use of the commutation algebra of the Gelfand basis. Starting with the product relation of the Gelfand matrices

$$\mathbf{E}_{m,n}\cdot\mathbf{E}_{i,k}=\delta_{n,i}\mathbf{E}_{m,k}(S12)$$

The possible commutation relations of $\mathbf{E}_{n,m}$ and $\mathbf{E}_{i,k}$ can be categorised depending on the relations of the indices n,m,i and k.

$$[\mathbf{E}_{m,n},\mathbf{E}_{i,k}]=\begin{cases}0 & if\; n=i=k=m\\ \mathbf{E}_{m,m}-\mathbf{E}_{n,n} & if\;(n=i)\neq(k=m)\\ 0 & if\; n\neq i\; and\; k\neq m\\ \mathbf{E}_{m,k} & if\; n=i\; and\; k\neq m\\ -\mathbf{E}_{i,n} & if\; n\neq i\; and\; k=m\end{cases}\quad (S13)$$

The Taylor expansion of the factors of the product in equation (S11) is

$$\exp\left(g_{m,n}[\mathbf{E}_{m,n}]\right)\mathbf{E}_{i,k}=\left(\mathbf{E}_{i,k}+g_{m,n}[\mathbf{E}_{m,n},\mathbf{E}_{i,k}]+\frac{{g_{m,n}}^2}{2!}[\mathbf{E}_{m,n},[\mathbf{E}_{m,n},\mathbf{E}_{i,k}]]\ldots\right)(S14)$$

Substituting the commutation relations from equation (S13) into equation (S14) yields the following possible resolutions of the expansion, depending on the Gelfand operators' indices.

$$\exp\left(g_{m,n}[\mathbf{E}_{m,n}]\right)\mathbf{E}_{i,k}$$

$$=\begin{cases}\mathbf{E}_{i,k}+g_{m,n}\left(\mathbf{E}_{k,k}-\mathbf{E}_{i,i}\right)-{g_{m,n}}^2\mathbf{E}_{k,i} & k=m\quad i=n\quad n\neq m\quad i\neq k\\ \mathbf{E}_{i,k}-g_{m,n}\mathbf{E}_{m,n} & k=m\quad i\neq n\quad n\neq m\quad i=k\\ \mathbf{E}_{i,k}-g_{m,n}\mathbf{E}_{i,n} & k=m\quad i\neq n\quad n\neq m\quad i\neq k\\ \mathbf{E}_{i,k}+g_{m,n}\mathbf{E}_{m,n} & k\neq m\quad i=n\quad n\neq m\quad i=k\\ \mathbf{E}_{i,k}+g_{m,n}\mathbf{E}_{m,k} & k\neq m\quad i=n\quad n\neq m\quad i\neq k\\ e^{g_{m,n}}\mathbf{E}_{i,k} & k\neq m\quad i=n\quad n=m\quad i\neq k\\ e^{-g_{m,n}}\mathbf{E}_{i,k} & k=m\quad i\neq n\quad n=m\quad i\neq k\\ \mathbf{E}_{i,k} & else\end{cases}$$

$$(S15)$$

By repeatedly drawing the formulas in equation (S15), the product on the LHS of equation (S11) can be turned into a sum of Gelfand matrices with coefficients.

$$\left(\prod_{i,k=-j}^{j} e^{g_{i,k}[\mathbf{E}_{i,k'}]}\right)\mathbf{E}_{m,m} = \sum_{i,k} \vartheta_{i,k}(\{g_{m,n}\})\,\mathbf{E}_{i,k} \quad (S16)$$

In this way, the whole LHS of equation (S11) can be written as a sum over Gelfand matrices.

$$\sum_{i,k} \phi_{i,k}(\{g_{m,n}\})\,\mathbf{E}_{i,k} = \sum_{k} \lambda_k(\{g_{m,n}\})\,\mathbf{X}_k \quad (S17)$$

This is equation (18) of the main paper.

Section 3: The perturbation used in the numerical results section of the main text

Figure (S1) shows the perturbation, $f(t) = f/\cosh\left(\frac{t-t_0}{\tau}\right)$, with parameters $f = 9\times10^{-5}$ $a.u.$, $\tau = 240\ a.u.$ and $t_0 = 2000\ a.u.$ This is the perturbation used to produce the numerical results in the main text and section 4 of this SI.

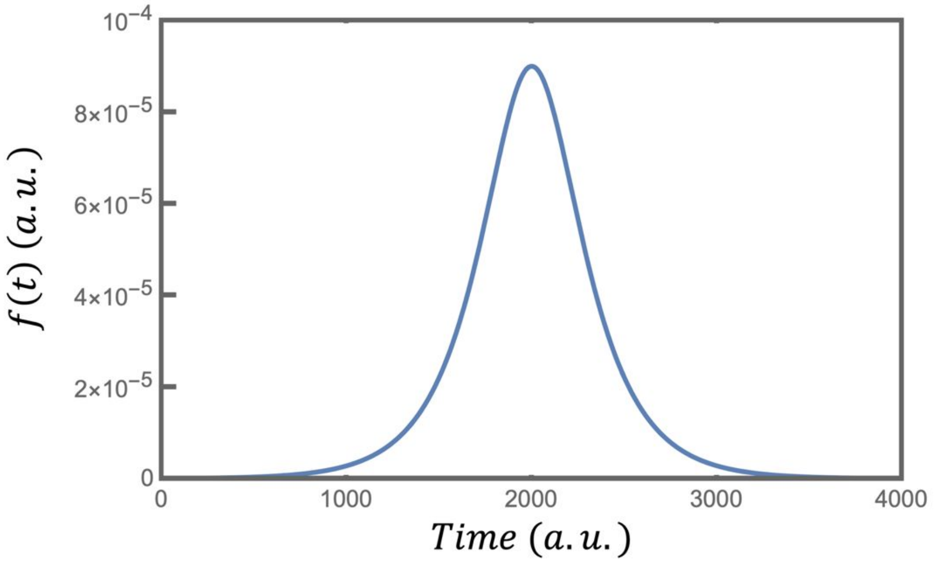

Figure S1: Force used to perturb the anharmonic oscillator and produce the numerical results in the main text and section 4 of this SI.

Section 4: Fidelities of the reconstructed density matrices of maximal entropy, with once and twice compacted surprisals, to the uncompressed exact density matrix

Figure (S2) shows the fidelities of the compressed density matrices of maximal entropy, $\boldsymbol{\rho}^{ME}$, to the original, uncompressed density matrix calculated with the Liouville-von Neumann equation (equation (30) of the main text), $\boldsymbol{\rho}^{LvN}$. The fidelities of two compressions are shown. As the fidelities is so close to one, a measure of one minus the fidelity, $1-F(\boldsymbol{\rho}^{LvN},\boldsymbol{\rho}^{ME})$, is used on the ordinate of figure (S2) to enable on to better see the results. The two compressions shown are equations (24) and (29) of the main text, compressing the surprisals of the $\boldsymbol{\rho}^{ME}$ to $3j-1$ and j constraints, respectively. The fidelities are calculated using $F(\boldsymbol{\rho}^{LvN},\boldsymbol{\rho}^{ME}) = tr\left(\sqrt{\sqrt{\boldsymbol{\rho}^{ME}}\,\boldsymbol{\rho}^{LvN}\sqrt{\boldsymbol{\rho}^{ME}}}\right)$.

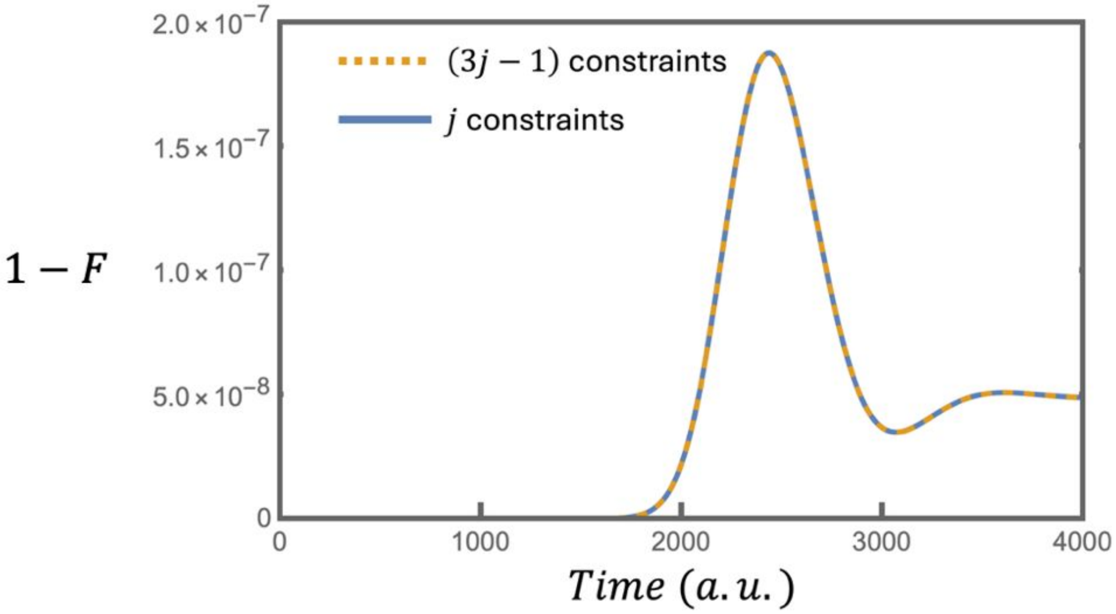

Figure S2: The fidelities of two compressions are compared using the measure of $1-F(\boldsymbol{\rho}^{LvN},\boldsymbol{\rho}^{ME})$ on the ordinate. The yellow dashed line shows $1-F$ of the fidelity of the compressed $\boldsymbol{\rho}^{ME}$ with $3j-1$ constraints in the surprisal (equation (24) of the main text). The blue line shows $1-F$ of the fidelity of the compressed $\boldsymbol{\rho}^{ME}$ with j constraints in the surprisal (equation (29) of the main text).

The shape of Figure (S2) is the same as that of Figure (1) of the main text, which shows the entropy difference of the compressed and uncompressed data, ΔS. Indeed, the two figures look identical but for the scale of the ordinate.

This can be shown numerically by taking the ratio of the two figures at times after the pulse. Before and at the beginning of the pulse, numerical noise greatly affects the ratios.

$$\frac{\Delta S(t)}{\Delta F(t)} \cong 7.15 \;\; \forall \;\; t > t_0 \; (S18)$$

Section 5: An extended elementwise comparison of an exactly time-propagated density matrix with a density matrix of maximum entropy that has a compacted surprisal

In the main text, we provided two figures comparing the ground state population, and ground-first excited state coherence, of the uncompacted, exact density matrix, $\boldsymbol{\rho}^{LvN}$, with those of the compacted density matrix of maximal entropy, $\boldsymbol{\rho}^{ME}$. $\boldsymbol{\rho}^{LvN}$ was calculated from the Liouville-von Neumann equation (equation (30) of the main text) and $\boldsymbol{\rho}^{ME}$ was constructed with j constraints in the surprisal (equation (29) of the main text).

In this section of the SI, we provide a further elementwise comparison of these $\boldsymbol{\rho}^{ME}$ and $\boldsymbol{\rho}^{LvN}$ for excited state populations (figures (S3) to (S7)) and coherences between the ground and higher excited states (figures (S8) to (S11)).

As is stated in the main text, these data are calculated for an anharmonic oscillator which is a 33-state molecule ($j = 32$) with parameter $A = 20\ cm^{-1}$ in equation (1) of the main text. The temperature of the system is $T = 1000\ K$, therefore giving a value of $\beta = 315.6\ a.u.$, which defines the initial unperturbed thermal equilibrium population distribution. This oscillator is perturbed by the force shown in figure (S1).

Figure (2) of the main text, and figures (S3) to (S7), show that the compacted density matrix of maximal entropy reproduces the populations of the ground and lower energy states better than the higher excited states. This is as expected, and a quick way to see this is to consider the entropy of the distribution of populations $S = -\sum_i \rho_{ii} \ln \rho_{ii}$. Seek the maximum of S

subject to the constraints $\sum_k \lambda_k \sum_i \rho_{ii} Y_{ii}^k$. By imposing the constraints, we can freely vary the populations ρ_{ii} so as to get the maximum $\delta S - \delta \sum_k \lambda_k \sum_i \rho_{ii} Y_{ii}^k = -\sum_i \delta\rho_{ii}(\ln \rho_{ii} - \sum_k \lambda_k Y_{ii}^k)$. The larger the population the more it contributes to the variation.

Figure (3) of the main text, and figures (S8) to (S11), show that the compaction reproduces the coherences between the ground and first excited state very well, but that the reproduction becomes less good for the coherences between the ground and higher excited state. This pattern is the same for coherence between the first excited state and the second, third, fourth… excited states etc… The compaction recovers the coherence between nearest neighbours very well, but the reproduction is less good as the spacing between the states in coherent superposition increases. This is a consequence of the specific structure of the set of constraints which was used in the surprisal of $\boldsymbol{\rho}^{ME}$. The constraints, equation (23) of the main text, were specifically chosen because they contained the information about nearest-neighbour coupling. For this reason, the $\boldsymbol{\rho}^{ME}$ constructed with this set of constraints in the compacted surprisal reproduces the coherences between nearest neighbours excellently, but between more distant neighbours less well.

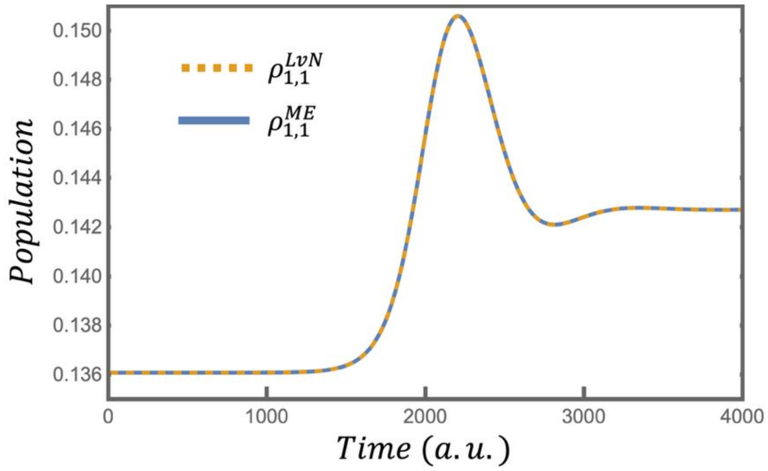

Figure S3: Comparison of the first excited state populations of the compacted $\boldsymbol{\rho}^{ME}$ (blue line) and the exact $\boldsymbol{\rho}^{LvN}$(yellow dashed line)

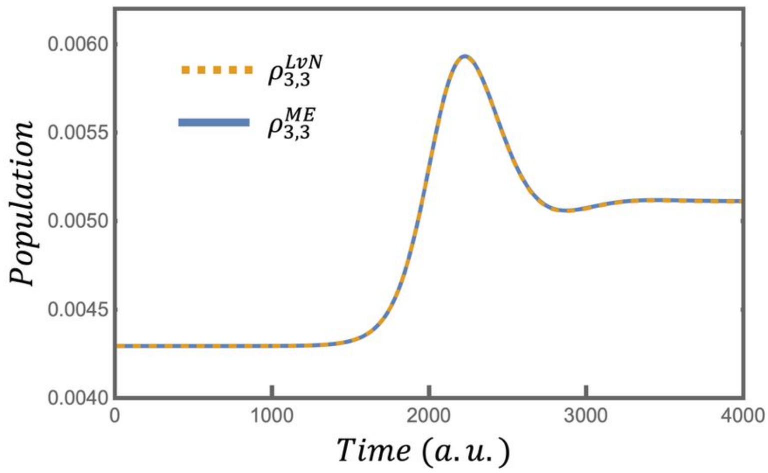

Figure S4: Comparison of the third excited state populations of the compacted $\boldsymbol{\rho}^{ME}$ (blue line) and the exact $\boldsymbol{\rho}^{LvN}$(yellow dashed line)

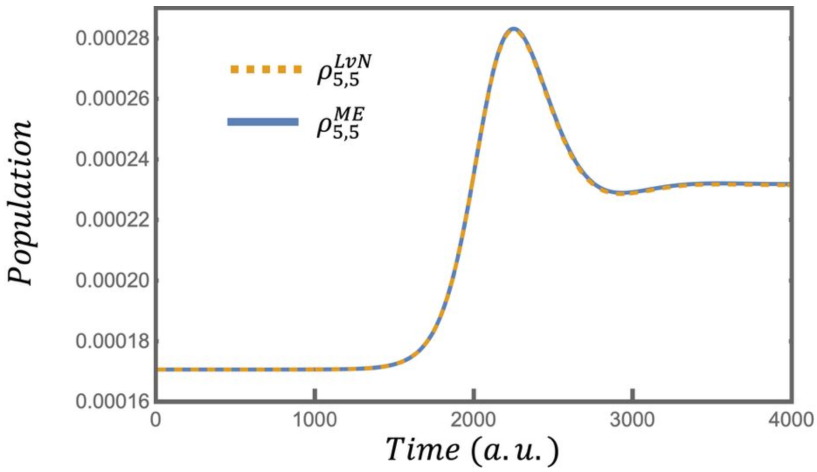

Figure S5: Comparison of the fifth excited state populations of the compacted $\boldsymbol{\rho}^{ME}$ (blue line) and the exact $\boldsymbol{\rho}^{LvN}$(yellow dashed line)

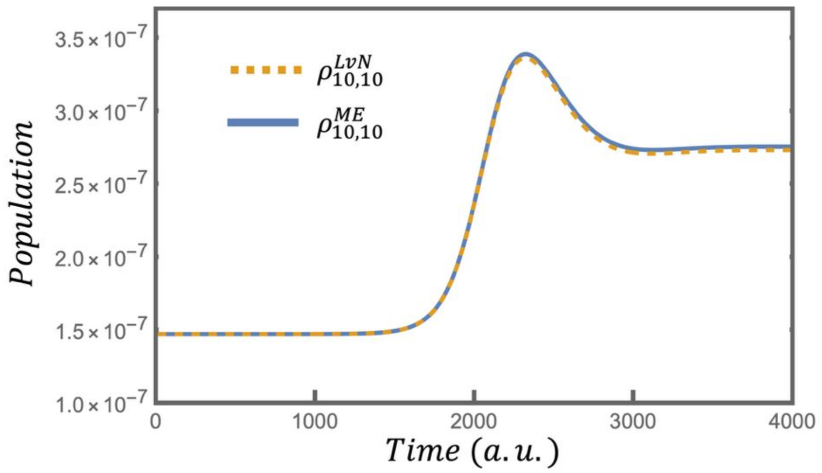

Figure S6: Comparison of the tenth excited state populations of the compacted $\boldsymbol{\rho}^{ME}$ (blue line) and the exact $\boldsymbol{\rho}^{LvN}$ (yellow dashed line)

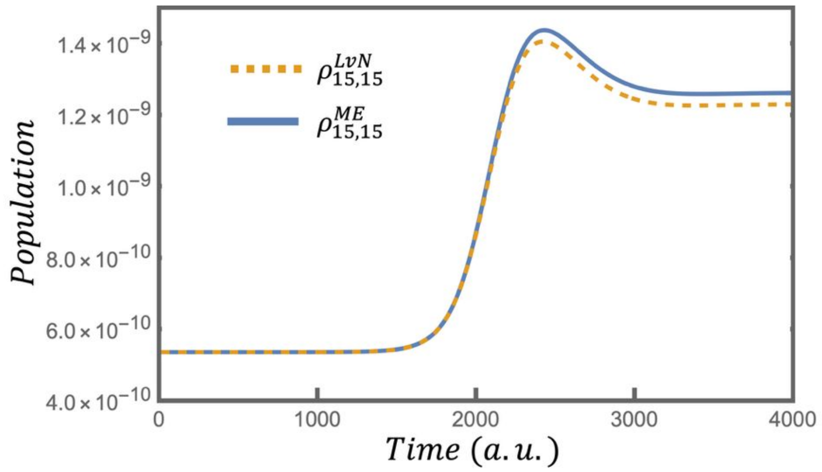

Figure S7: Comparison of the fifteenth excited state populations of the compacted $\boldsymbol{\rho}^{ME}$ (blue line) and the exact $\boldsymbol{\rho}^{LvN}$(yellow dashed line)

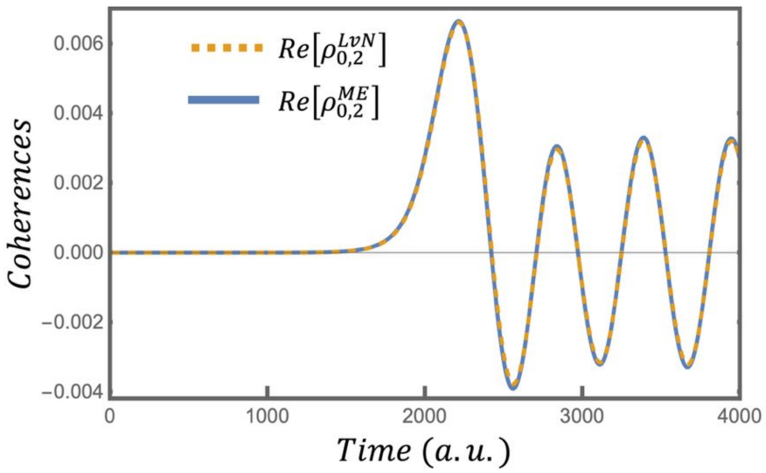

Figure S8: Comparison of the real part of the coherence between the ground and second excited states of the compacted $\boldsymbol{\rho}^{ME}$ (blue line) and the exact $\boldsymbol{\rho}^{LvN}$ (yellow dashed line)

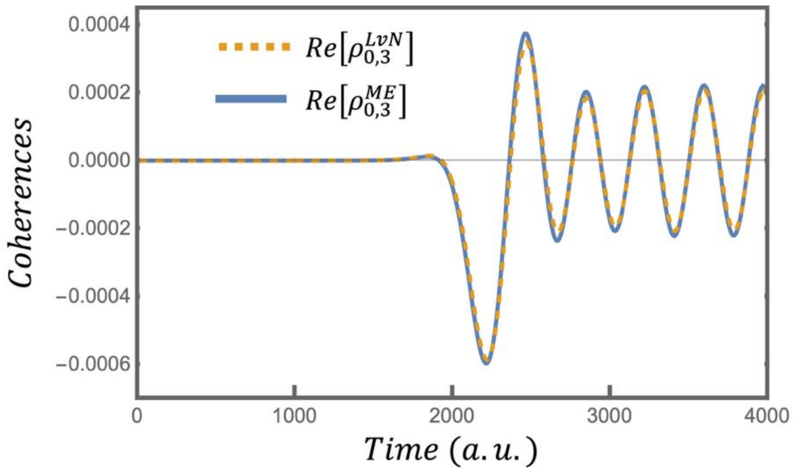

Figure S9: Comparison of the real part of the coherence between the ground and third excited states of the compacted $\boldsymbol{\rho}^{ME}$ (blue line) and the exact $\boldsymbol{\rho}^{LvN}$(yellow dashed line)

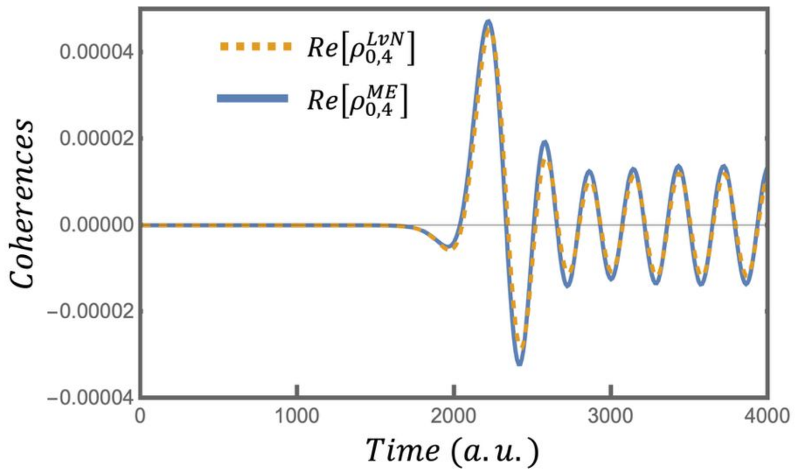

Figure S10: Comparison of the real part of the coherence between the ground and fourth excited states of the compacted $\boldsymbol{\rho}^{ME}$ (blue line) and the exact $\boldsymbol{\rho}^{LvN}$(yellow dashed line)

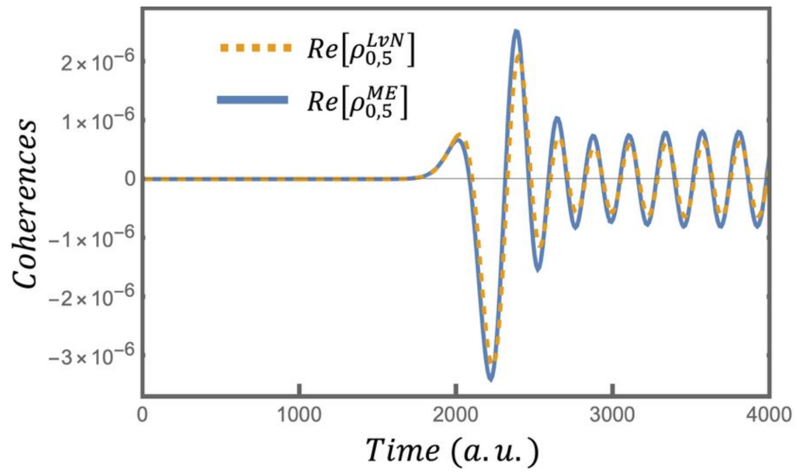

Figure S11: Comparison of the real part of the coherence between the ground and fifth excited states of the compacted $\boldsymbol{\rho}^{ME}$ (blue line) and the exact $\boldsymbol{\rho}^{LvN}$(yellow dashed line)

Section 6: An explicit analytical derivation of the Lagrange parameters of a system with a small $\boldsymbol{j} = \mathbf{1}$ molecule

This section provides an explicit analytic derivation of the Lagrange parameters in the surprisal, for Gelfand constraints. These Lagrange parameters are derived in terms of the group parameters of the factorised unitary time evolution operator, constructed with a Lie algebra of

Gelfand matrices. Equations of motion for these group parameters are derived in section 1 of the SI.

For $j = 1$, equation (17) of the main text can be written out explicitly.

$$(\mathcal{Z} - 2\beta \mathrm{A}) \sum_{m=-1}^{1} \mathbf{E}_{m,m} + \beta \mathrm{A} \left(\prod_{i,k=-1} e^{g_{i,k}[\mathbf{E}_{i,k'}]} \right) \mathbf{E}_{-1,-1} + \beta \mathrm{A} \left(\prod_{i,k=-j}^{j} e^{g_{i,k}[\mathbf{E}_{i,k'}]} \right) \mathbf{E}_{1,1} = -i \sum_{k} \lambda_k \mathbf{X}_k$$

$$(S19)$$

Using the rules in equation (S15), the products of the LHS of this equation can be written out explicitly.

$$\beta \mathrm{A} \left(\prod_{i,k=-j}^{j} e^{g_{i,k}[\mathbf{E}_{i,k'}]} \right) \mathbf{E}_{-1,-1}$$

$$= \beta \mathrm{A}\big(1 + g_{1,-1} g_{-1,1} + g_{-1,0}\big(g_{0,-1} + g_{1,-1} g_{0,1} e^{g_{0,0}}\big)\big)\mathbf{E}_{-1,-1}$$

$$- \beta \mathrm{A} g_{-1,0}\big(g_{-1,0}\big(g_{0,-1} + g_{1,-1} g_{0,1} e^{g_{0,0}}\big) + \big(1 + g_{1,-1} g_{-1,1}\big)\big) e^{g_{-1,-1}} \mathbf{E}_{-1,0}$$

$$- \beta \mathrm{A} g_{-1,1}\big(1 + g_{1,-1} g_{-1,1} + g_{-1,0}\big(g_{0,-1} + g_{1,-1} g_{0,1} e^{g_{0,0}}\big)\big) e^{g_{-1,-1}} \mathbf{E}_{-1,1} + \beta \mathrm{A}\big(g_{0,-1} + g_{1,-1}$$

$$- \beta \mathrm{A} g_{-1,0}\big(g_{0,-1} + g_{1,-1} g_{0,1} e^{g_{0,0}}\big)\mathbf{E}_{0,0} - \beta \mathrm{A}\big(g_{0,-1} + g_{1,-1} g_{0,1} e^{g_{0,0}}\big) g_{-1,1} \mathbf{E}_{0,1}$$

$$+ \beta \mathrm{A} g_{1,-1} e^{-g_{-1,-1}} \mathbf{E}_{1,-1} - \beta \mathrm{A} g_{1,-1} g_{-1,0} \mathbf{E}_{1,0} - \beta \mathrm{A} g_{1,-1} g_{-1,1} \mathbf{E}_{1,1}$$

$$(S20)$$

and

$$\beta \mathrm{A}\left(\prod_{i,k=-j}^{j} e^{g_{i,k}[\mathbf{E}_{i,k},]}\right)\mathbf{E}_{1,1}$$

$$= \beta \mathrm{A}\big(\big(g_{1,0}g_{0,-1}e^{-g_{0,0}} - g_{1,-1}\big)g_{-1,1} + g_{-1,0}g_{0,1}\big(g_{1,0}g_{0,-1} - g_{1,-1}e^{g_{0,0}}\big)\big)\mathbf{E}_{-1,-1}$$

$$- \beta \mathrm{A}\big(\big(g_{1,0}g_{0,-1}e^{-g_{0,0}} - g_{1,-1}\big)g_{-1,1}g_{-1,0} + g_{1,0}e^{-g_{0,0}}g_{-1,1}$$

$$+ g_{-1,0}{}^{2}g_{0,1}\big(g_{1,0}g_{0,-1} - g_{1,-1}e^{g_{0,0}}\big) + g_{1,0}g_{0,1}g_{-1,0}\big)e^{g_{-1,-1}}\mathbf{E}_{-1,0}$$

$$+ \beta \mathrm{A}\big(g_{-1,1}\big(1 + g_{1,0}g_{0,1} - \big(g_{1,0}g_{0,-1}e^{-g_{0,0}} - g_{1,-1}\big)g_{-1,1}\big)$$

$$+ \big(\big(g_{1,0}g_{0,1} + 1\big)g_{0,1}e^{g_{0,0}} - g_{-1,1}g_{0,1}\big(g_{1,0}g_{0,-1} - g_{1,-1}e^{g_{0,0}}\big)\big)g_{-1,0}\big)e^{g_{-1,-1}}\mathbf{E}_{-1,1}$$

$$+ \beta \mathrm{A}g_{0,1}\big(g_{1,0}g_{0,-1} - g_{1,-1}e^{g_{0,0}}\big)e^{-g_{-1,-1}}\mathbf{E}_{0,-1}$$

$$- \beta \mathrm{A}g_{0,1}\big(g_{1,0} + g_{-1,0}\big(g_{1,0}g_{0,-1} - g_{1,-1}e^{g_{0,0}}\big)\big)\mathbf{E}_{0,0}$$

$$+ \beta \mathrm{A}\big(\big(g_{1,0}g_{0,1} + 1\big)g_{0,1}e^{g_{0,0}} - g_{-1,1}g_{0,1}\big(g_{1,0}g_{0,-1} - g_{1,-1}e^{g_{0,0}}\big)\big)\mathbf{E}_{0,1} + \beta \mathrm{A}\big(g_{1,0}g_{0,-1}e^{-g_{0,}}$$

$$- \beta \mathrm{A}\big(g_{-1,0}\big(g_{1,0}g_{0,-1}e^{-g_{0,0}} - g_{1,-1}\big) + g_{1,0}e^{-g_{0,0}}\big)\mathbf{E}_{1,0} + \beta \mathrm{A}\big(1 + g_{1,0}g_{0,1} - \big(g_{1,0}g_{0,-1}e^{-g_{0}}$$

$$(S21)$$

By defining the basis on the RHS of equation (S19) as the Gelfand matrices, $\{\mathbf{X}_i\} = \{\mathbf{E}_{i,k}\}$, equation (S17) becomes $\sum_{i,k} \phi_{i,k}(\{g_m\})\,\mathbf{E}_{i,k} = -i\sum_{i,k} \lambda_{i,k}(\{g_m\})\,\mathbf{E}_{i,k}$. Therefore,

$$i\mathbf{\Phi} = \mathbf{\Lambda}\ (S22)$$

For a general system of $j+1$ bound states

$$\mathbf{\Lambda} = \begin{pmatrix} \lambda_{-j,-j}(\{g_m\}) & \lambda_{-j,-j+1}(\{g_m\}) & \cdots & \cdots & \lambda_{-j,j}(\{g_m\}) \\ \lambda_{-j+1,-j}(\{g_m\}) & \ddots & \ddots & \lambda_{-1,1}(\{g_m\}) & \vdots \\ \vdots & \ddots & \lambda_{0,0}(\{g_m\}) & \lambda_{0,1}(\{g_m\}) & \ddots \\ \vdots & \lambda_{1,-1}(\{g_m\}) & \lambda_{1,0}(\{g_m\}) & \ddots & \lambda_{j-1,j}(\{g_m\}) \\ \lambda_{j,-j}(\{g_m\}) & \cdots & \ddots & \lambda_{j,j-1}(\{g_m\}) & \lambda_{j,j}(\{g_m\}) \end{pmatrix} (S23)$$

For $j = 1$, using the above equation, the Lagrange parameter corresponding to the Gelfand constraints are

$$\lambda_{-1,-1} = i\mathcal{Z} - i\beta \mathrm{A}\big(1 - g_{-1,0}g_{0,-1} - g_{-1,0}g_{0,1}g_{1,0}g_{0,-1} - g_{1,0}g_{0,-1}g_{-1,1}e^{-g_{0,0}}\big)$$

$$\lambda_{-1,0} = -i\beta \mathrm{A}\left(g_{-1,0}g_{0,-1} + 1\right)\left(g_{1,0}g_{0,1}g_{-1,0} + g_{-1,0} + g_{1,0}g_{-1,1}e^{-g_{0,0}}\right)e^{g_{-1,-1}}$$

$$\lambda_{-1,1} = i\beta \mathrm{A}\left(g_{-1,1}g_{1,0}g_{0,1}\left(1 - g_{0,-1}g_{-1,0}\right) - g_{-1,1}g_{0,-1}\left(g_{-1,1}g_{1,0}e^{-g_{0,0}} + g_{-1,0}\right) + \left(g_{1,0}g_{0,1} + 1\right)g_{-1,0}g_{0,1}e^{g_{0,0}}\right)e^{g_{-1,-1}}$$

$$\lambda_{0,-1} = i\beta \mathrm{A}\left(1 + g_{0,1}g_{1,0}\right)g_{0,-1}e^{-g_{-1,-1}}$$

$$\lambda_{0,0} = i\mathcal{Z} - i\beta \mathrm{A}\left(1 + \left(1 + g_{-1,0}g_{0,-1}\right)\left(1 + g_{0,1}g_{1,0}\right)\right)$$

$$\lambda_{0,1} = i\beta \mathrm{A}\left(g_{0,1}e^{g_{0,0}} - g_{-1,1}g_{0,-1}\right)\left(g_{0,1}g_{1,0} + 1\right)$$

$$\lambda_{1,-1} = i\beta \mathrm{A}g_{1,0}g_{0,-1}e^{-g_{0,0}-g_{-1,-1}}$$

$$\lambda_{1,0} = -i\beta \mathrm{A}g_{1,0}\left(g_{-1,0}g_{0,-1} + 1\right)e^{-g_{0,0}}$$

$$\lambda_{1,1} = i\mathcal{Z} - i\beta \mathrm{A}\left(1 - g_{1,0}g_{0,1} + g_{1,0}g_{0,-1}g_{-1,1}e^{-g_{0,0}}\right)$$

$$(S24)$$

As stated previously, the $\{g_{q,p}\}$ in these equations are calculated using the equations of motion described in section 1 of the SI.

References SI

(1) Wei, J.; Norman, E. Lie Algebraic Solution of Linear Differential Equations. *Journal of Mathematical Physics* **1963**, *4* (4), 575-581. DOI: 10.1063/1.1703993 (acccessed 9/7/2025).

(2) Wei, J.; Norman, E. On Global Representations of the Solutions of Linear Differential Equations as a Product of Exponentials. *Proceedings of the American Mathematical Society* **1964**, *15* (2), 327-334. DOI: 10.2307/2034065 (acccessed 2025/09/07/).JSTOR.

(3) Guerrero, J.; Berrondo, M. Semiclassical interpretation of Wei–Norman factorization for SU(1, 1) and its related integral transforms. *Journal of Mathematical Physics* **2020**, *61* (8). DOI: 10.1063/1.5143586 (acccessed 9/7/2025).

(4) Hamilton, J. R.; Levine, R. D.; Remacle, F. Constructing Dynamical Symmetries for Quantum Computing: Applications to Coherent Dynamics in Coupled Quantum Dots. *Nanomaterials* **2024**, *14* (24), 2056.
(5) Altafini, C. Explicit Wei-Norman formulae for matrix Lie groups. In *Proceedings of the 41st IEEE Conference on Decision and Control, 2002.*, 10-13 Dec. 2002, 2002; Vol. 3, pp 2714-2719 vol.2713. DOI: 10.1109/CDC.2002.1184251.
(6) Hamilton, J. R.; Remacle, F.; Levine, R. D. Compacting the Time Evolution of the Forced Morse Oscillator Using Dynamical Symmetries Derived by an Algebraic Wei-Norman Approach. *Journal of Chemical Theory and Computation* **2025**, *21* (9), 4347-4356. DOI: 10.1021/acs.jctc.5c00148.
(7) Hall, B. C. *Lie Groups, Lie Algebras, and Representations: An Elementary Introduction*; Springer, 2015.
